# Materials Genomics Search for Possible Helium‐Absorbing Nano‐Phases in Fusion Structural Materials

**DOI:** 10.1002/advs.202203555

**Published:** 2022-09-30

**Authors:** Haowei Xu, So Yeon Kim, Di Chen, Jean‐Phillippe Monchoux, Thomas Voisin, Cheng Sun, Ju Li

**Affiliations:** ^1^ Department of Nuclear Science and Engineering Massachusetts Institute of Technology Cambridge MA 02139 USA; ^2^ Department of Materials Science and Engineering Massachusetts Institute of Technology Cambridge MA 02139 USA; ^3^ Department of Physics and Texas Center for Superconductivity University of Houston Houston TX 77204 USA; ^4^ Centre for Materials Elaboration and Structural Studies University of Toulouse French National Centre for Scientific Research Toulouse 31055 France; ^5^ Materials Science Division Lawrence Livermore National Laboratory Livermore CA 94550 USA; ^6^ Characterization and Advanced PIE Division Idaho National Laboratory Idaho Falls ID 83415 USA

**Keywords:** density functional theory calculations, fusion structural materials, high‐throughput screening

## Abstract

Civilian fusion demands structural materials that can withstand the harsh environments imposed inside fusion plasma reactors. The structural materials often transmute under 14.1 MeV fast neutrons, producing helium (He), which embrittles the grain boundary (GB) network. Here, it is shown that neutron‐friendly and mechanically strong nano‐phases with atomic‐scale free volume can have low He‐embedding energy $E_{\mathrm{emb}}$ and >10 at.% He‐absorbing capacity, and can be especially advantageous for soaking up He on top of resisting radiation damage and creep, provided they have thermodynamic compatibility with the matrix phase, satisfactory equilibrium wetting angle, as well as a high enough melting point. The preliminary experimental demonstration proves that $E_{\mathrm{emb}}$ is a good ab initio predictor of He shielding potency in nano‐heterophase materials, and thus, $E_{\mathrm{emb}}$ is used as a key feature for computational screening. In this context, a list of viable compounds expected to be good He‐absorbing nano‐phases is presented, taking into account $E_{\mathrm{emb}}$, the neutron absorption and activation cross‐sections, the elastic moduli, melting temperature, the thermodynamic compatibility, and the equilbrium wetting angle of the nano‐phases with the Fe matrix as an example.

## Graphical abstract

In fusion plasma reactors, helium (He) can be produced by fast‐neutron irradiation and deuterium–tritium (D–T) reaction, and embrittles grain boundaries (GBs). Combining experiments and computations, it is shown that neutron‐friendly and mechanically strong nano‐phases with atomic‐scale free volume could be advantageous for soaking up He, thereby protecting the matrix GBs from He attack. A list is presented for phases with such capabilities.

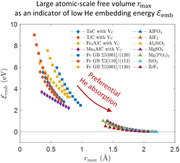

## 1 Introduction

Ever since harvesting nuclear fission energy for electricity production became a reality in 1957, fusion power has been the dream energy source for terrestrial applications and space explorations. To enable nuclear fusion, it is necessary to allow fast neutrons with kinetic energy as high as 14.1 MeV to impinge on the plasma vessel material. This poses a grand challenge to material scientists, since when bombarded by these high‐energy neutrons, transition metals—the primary constituent elements of the materials for structural components—often transmute to produce helium (He).^[^ ^1^ ^]^ Because He is an inert‐gas element, it interacts with the matrix atoms in a repulsive manner and thus tends to segregate into grain boundaries (GBs), which can have a somewhat larger free volume than the lattice.^[^ ^2^ ^]^ Once a monolayer of He (coverage of ≈7 × 10^14^ cm^−2^ or 7 nm^−2^)^[^ ^3^ ^]^ manages to segregate on a 2D GB, they jack up the metal–metal bonding distances across the GB to be sufficiently extended so that the GB can no longer sustain appreciable tensile or shear load, and would easily debond. To make matters worse, as a 2D GB cannot freely terminate crystallographically, most GBs are not alone but part of a percolating 2D GB network, which forms a natural template for long cracks that are highly damaging. 2D traction‐free openings are the worst form of damage; among spherical, prolate (needle‐shaped), and oblate (disk‐shaped) cavities in a solid body, the oblate‐shaped cavities engender the largest stress amplification factor that tends to infinity when the long‐to‐short axis ratio turns to infinity,^[^ ^4^ ^]^ thereby self‐evolving into a penny‐shaped crack. The stress amplification factors in prolate and spherical cavities, in contrast, stay bounded. GBs are thus naturally less tolerant against damage growth owing to their locally 2D free‐volume segregation and globally percolating topology.^[^ ^2^, ^5^ ^]^ By this mechanism, He in structural components can be extremely detrimental even with very low bulk concentration (≈0.01 at.%, or 100 appm), if they manage to diffuse to and segregate at the GBs.

In this regard, nanodispersion‐strengthened materials, which were originally developed for high‐temperature creep‐resistance,^[^ ^6^ ^]^ have drawn attention as fusion structural materials with their remarkable radiation resistance (measured in the unit of displacements per atom, dpa) and He tolerance (measured in the unit of atomic ppm of He per matrix atom, appm).^[^ ^1^, ^7^, ^8^ ^]^ In particular, oxide‐dispersion‐strengthened (ODS) steels have been demonstrated to have tolerance against He‐induced embrittlement as well as superior tensile, creep, and fatigue strength at elevated temperatures.^[^ ^9^ ^]^ Transmission electron microscopy (TEM) studies have revealed that He bubbles and voids form preferentially in the close vicinity to the nano‐oxide dispersions in ODS steels.^[^ ^10^ ^]^ The phase boundaries (PBs), e.g., oxide‐matrix interfaces, can be sinks for He because they are often incoherent interfaces with excess atomic‐scale free volume. But unlike the GBs of a polycrystal, PBs wrapping the nanodispersoids are topologically different in that they are isolated and do not form a percolating network, as the second phase volume fraction is often only on the order of 1 vol.%, below the percolation threshold. And thus even when the PB around an isolated nanoparticle becomes fully debonded after radiation, the limited spatial extent of ≈10 nm of such opening and the sphericity of the nanoparticle mean such an opening around the “0D” nano‐phase is much less damaging, with non‐singular stress‐amplification factor independent of the spherical diameter. The same can be shown for “1D” or needle/wire‐like nano‐phases—even their complete debonding with the matrix does not lead to singularity in the stress‐amplification factor, regardless of the needle orientation with respect to stress. Thus, uniformly dispersed “0D”/“1D” morphology nano‐heterophases may screen and shield the GB from He segregation by attracting and soaking up the He inside their own lattices or at the incoherent PBs, if these sites have lower He embedding energy ($E_{\mathrm{emb}}$) than the percolating matrix GBs. In this work, we propose that by dispersing nanoparticles or nanowires of secondary phases (that may not be limited to oxides but could also be carbides, nitrides, borides, etc.), the lattice interior of such secondary phases may absorb He atoms with significantly lower embedding energy than that of the matrix GB, thereby screening and protecting the matrix GBs from He attack.

For the proposed He‐tolerant design, let us first examine some numbers in practice. We note that the engineering requirement is often to tolerate a few thousand appm He—in the fusion reactor material, the appm‐to‐dpa ratio is typically ≈10, thus a desirable fusion structural material should be able to sustain a few hundred dpa radiation damage and a few thousand appm He, at temperatures up to 800 °C. This would allow a few years of service life for the ARC (affordable, robust, compact) power reactor vacuum vessel.^[^ ^11^ ^]^ Previously, we have already demonstrated uniform dispersion of 1D carbide nano‐phases at up to 2 vol.% can greatly improve room‐temperature tensile strength as well as high‐temperature creep strength without sacrificing the tensile ductility.^[^ ^5^, ^12^, ^13^ ^]^ The system also showed superior radiation resistance up to 70 dpa.^[^ ^12^ ^]^ This is attributed to the 1D carbide nano‐phases being radiation defect sinks.^[^ ^14^ ^]^ If such uniformly dispersed carbides, oxides, nitrides, phosphides, borides (^11^B), etc. can also absorb He within their bulk lattices, then even 10 at.% uptakes in the nano‐phase lattice should be able to soak up all the He in the matrix and protect the matrix GBs, since a few thousand appm can be accommodated by ≈10 at.% (uptakes per 2nd phases) × 2 vol.% (the volume fraction of 2nd phases in a composite), provided that $E_{\mathrm{emb}}$ (2nd phase) < $E_{\mathrm{emb}}$ (matrix GB) and that the nano‐heterophases (probably a few nanometre to 10^1^ nm in diameter) are uniformly distributed spatially when viewed at a 10^2^ nm length scale, which needs to be smaller than the matrix grain size to protect all areas of the GB from He segregation and attack.

In this work, we perform a systematic screening of crystals listed in the Materials Project database^[^ ^15^ ^]^ and show the top candidates for the nano‐phases with potentially low $E_{\mathrm{emb}}$ and decent He absorbing capacity (10 at.% or more), which could be advantageous for soaking up and delaying He damage on fusion structural materials. The screening criteria used are 1) low neutron absorption and activation, 2) relatively large elastic moduli, and 3) atomic‐scale free volume that can accommodate one or more He clusters, such as “constitutional vacancies” in some crystal lattices.^[^ ^16^ ^]^ To that end, we first illustrate that atomic‐scale free volume/pores are hallmarks of low $E_{\mathrm{emb}}$. Then combining experiments and calculations, we demonstrate that $E_{\mathrm{emb}}$ is, in turn, a good ab initio predictor for our He diversion strategy in order to avoid He damage. We study a two‐phase alloy consisting of TiAl (*γ*) and Ti_3_Al (*α*_2_) nanolamellas. Density functional theory (DFT) calculations show that the *α*_2_ phase has significantly lower $E_{\mathrm{emb}}$ than the *γ* phase. Experimental He ion implantation and post‐mortem TEM characterizations then show that indeed only the *α*_2_ phase is significantly damaged with He bubbles, while the adjacent *γ* phases appear very much “immune” from damage. This experimental validation sets the theoretical basis for our nano‐phase selection based on ab‐initio computed $E_{\mathrm{emb}}$.

There are also important auxiliary considerations in addition to the low $E_{\mathrm{emb}}$ and decent He absorbing capacity of the nano‐phases. First, the constituent atoms of the nano‐phases must be “neutron‐friendly”. That is, they should not cause too much excess neutron absorption/activation in order to not complicate the neutronics of the reactor or waste disposal, or lose the He‐binding effect over time. Second, the nano‐phases need to be mechanically strong in tension and shear to be able to sustain significant load locally. That is, they need to still serve as the strengthening phase for creep resistance.^[^ ^5^, ^12^, ^13^ ^]^ This is especially true if $E_{\mathrm{emb}}$ (2nd phase) < $E_{\mathrm{emb}}$ (PB) and thus the PB does not debond first. Third, the fusion reactor vessel will be operating at temperatures up to 800 °C, and thus the nano‐phases need to be thermally and chemically stable in contact with the matrix phase and be coarsening‐resistant. Thus the solubility and diffusivity of some of the second phase's constituent elements in the matrix phase should be very low, like O, N, etc., in the same way as ODS. Finally, in order to achieve uniform dispersion without aggregation during the processing of such structural nanocomposite, the nano‐phases also need to have a low enough wetting angle with the metal matrix. Herein, we will demonstrate the down‐selection of He‐absorbing nano‐phases, taking into account the melting temperature (*T*_M_), the thermodynamic compatibility, and the wetting angle of the nano‐phases with Fe matrix as an example.

## 2 Results

### 2.1 He‐Embedding Energy in Lattice Interior and GB of Fe, Half‐Heusler Phases, and Others

Before doing the high‐throughput computational screening, one needs to define and validate numerical metrics of high He absorbing capability. As we will elaborate on later, low He embedding energy $E_{\mathrm{emb}}$ (unit eV/He) can be a figure of merit. However, it is computationally demanding to do high‐throughput calculations on embedding energies, which require relatively expensive supercell calculations. Here, we propose that the atomic‐scale free volume can be an indicator of low embedding energy, and thus can serve as an “easy‐to‐compute” metric of good He‐absorbing nano‐phases. In the following, we will show that this assumption is verified by ab initio calculations. Since we will study He formation energy in various geometries in the following, we adopt the general term “embedding” energy rather than “interstitial formation” energy. The embedding energy is defined as

(1) $$E_{\mathrm{emb}}\left(m\right)\equiv E_{{\mathrm{He}}_{m}}^{\mathrm{tot}}-E_{{\mathrm{He}}_{0}}^{\mathrm{tot}}-mE_{\mathrm{He}},E_{\mathrm{emb}}\left(m\right)\equiv E_{\mathrm{emb}}/m$$

where *m* is the number of He atoms, $E_{{\mathrm{He}}_{m}}^{\mathrm{tot}}$ is the total energy of the system with *m* He atoms, $E_{{\mathrm{He}}_{0}}^{\mathrm{tot}}$ is the total energy of the system without He atoms (with other atoms the same), while *E*_He_ is the energy of a single isolated He atom, which is 89 meV in our supercell calculation setup.

**Figure 1:**
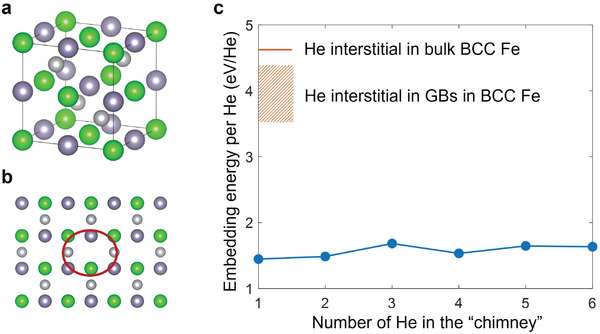
Presence of atomic‐scale free volume and He embedding energy. a) Atomic structure of ZrNiSn Half‐Heusler compound with constituent vacancies. b) The “free‐volume chimney” in ZrNiSn along [110] direction. c) $E_{\mathrm{emb}}$ of He atom in ZrNiSn (blue curve with dots), versus $E_{\mathrm{emb}}$ of He interstitial in various sites in BCC Fe. $E_{\mathrm{emb}}$ of He interstitial in GBs in BCC Fe depends on the local environment (distance from the GB, structure of the GB, etc.), and thus has a distribution, represented by the thick bar.

Previously, Erhart et al.^[^ ^17^ ^]^ comprehensively investigated He interstitial formation and migration energies in various oxides, including Al_2_O_3_, TiO_2_, Y_2_O_3_, (Mg, Ca, Sr, Ba)O, Y*_x_* Al*_y_* O*_z_*, via DFT calculations. They found that the solubility of He in oxides scales with the free volume at the interstitial site and is virtually independent of the oxide's chemical composition. Similar features are found in body‐centered cubic (BCC) Fe as well. In Ref. [18], $E_{\mathrm{emb}}$ for the octahedral and tetrahedral interstitial sites of BCC Fe are reported to be 4.60 and 4.37 eV/He, respectively, based on DFT calculations.^[^ ^18^ ^]^ On the other hand, when He is associated with a Fe vacancy, which provides a larger free volume, the embedding energy $E_{\mathrm{emb}}$ would decrease to ≈4 eV/He. Moreover, when He atoms reside in the GBs of BCC Fe, which have greater free volume, $E_{\mathrm{emb}}$ is found to be even lower by ≈1 eV/He, providing a strong incentive for GB segregation (**Figure** **1**c) and subsequent debonding damage, though the exact value depends on the angle and sigma value of the boundaries.^[^ ^18^ ^]^ These results suggest that $E_{\mathrm{emb}}$ is sensitive to the local atomic geometry, and particularly, $E_{\mathrm{emb}}$ would be lower when the local free volume is large. This point is further corroborated by Half‐Heusler (HH) phase ZrNiSn. The atomic structure of the HH phase features an extended “free volume chimney” infinitely long in a perfect crystal (Figure 1a,b). Indeed, our DFT calculations indicate that $E_{\mathrm{emb}}$ is on the order of just 1.5 eV/He in ZrNiSn, and $E_{\mathrm{emb}}$ per He does not increase much when multiple He atoms are put in the free‐volume chimney (Figure 1c) of this crystal. This corroborates that the key to reducing $E_{\mathrm{emb}}$ is to provide a spacious room for He atoms. While the HH phases serve as an illustration of low $E_{\mathrm{emb}}$ and decent He absorbing capacity, the ZrNiSn compound itself is not very neutron‐friendly, as Ni and Sn will have high long‐term activity after exposure to fast neutrons, thus presenting a nuclear waste problem. Later, we will systematically show that the atomic‐scale free volume is a good indicator of low $E_{\mathrm{emb}}$ with first‐principle calculations.

Below, combining experiments and DFT calculations, we show that the raw magnitude of $E_{\mathrm{emb}}$ is the key feature for the strategy of He diversion and screening, and offers protection of certain phases even when the overall He injection exceeds 10^4^ appm, way more than the level typically required for a civilian fusion reactor. Subsequently, we propose that atomic‐scale free volume, which indicates low $E_{\mathrm{emb}}$, would be a good predictor of strong He absorbing capability. Based on this rationale, we perform large‐scale computational screening for potential He‐absorbing nano‐phases, considering also the processability, load‐bearing ability, and neutronics aspects of fusion reactors.

### 2.2 Helium Diversion and Screening, Damage Avoidance in Two‐Phase Ti–Al Alloy

**Figure 2:**
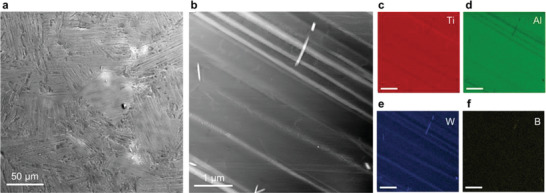
Microstructural characterization of as‐fabricated Ti‐48Al‐2W‐0.08B (at.%) alloy with *γ* (TiAl) and *α*_2_ (Ti_3_Al) nano‐lamellae. a) SEM micrograph showing equiaxed grains with interior nano‐lamellar structures. b) STEM micrograph showing the two phases with *γ* phases in dark contrast and *α*_2_ phase in bright contrast. c–f) EDS mapping of the major chemical composition of Ti and Al, and minor elements of W and B. Scale bars in (c–f), 1 µm.

**Figure** **2** shows the characterization of the as‐fabricated Ti‐48Al‐2W‐0.08B (at.%) alloy with a nano‐lamellar heterophase structure using scanning electron microscopy (SEM) and scanning transmission electron microscopy (STEM). Equiaxed grains with interior nano‐lamellar structures were clearly identified in the SEM micrograph (Figure 2a). The two‐phase components (*γ* and *α*_2_) in the grain interiors were distinguished in the STEM micrograph with the *γ* phase in the dark contrast and *α*_2_ in the bright contrast, as shown in Figure 1b. Three types of interfaces (*γ*/*γ*, *γ*/*α*_2_, and *α*_2_/*α*_2_) were observed in the as‐fabricated alloy. The chemistry analysis with electron dispersive spectroscopy (EDS) mapping suggests that minor element tungsten (W), which was added to improve mechanical properties, preferentially segregates to the *α*_2_ phase, while depletes in the *γ* phase; the segregation of W did not change the crystal structure of the *α*_2_ phase (Figure S1, Supporting Information).

**Figure 3:**
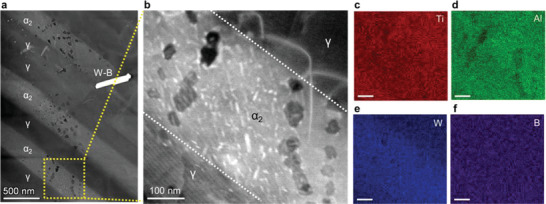
Characterization of He‐irradiated Ti‐48Al‐2W‐0.08B alloy at 750 °C with 10000 appm He. a) STEM micrograph showing the peak damage region of Ti‐48Al‐2W‐0.08B alloy. b) Enlarged view of the boxed region in (a). A high density of He gas bubbles (≈2 × 10^22^ m^−3^) form in the *α*_2_ phase, while a much lower density of He gas bubbles forms in the *γ* phase. (c–f) EDS map showing no evidence of chemical segregation in the vicinity of He bubbles and interfaces. Scale bars in (c–f), 100 nm.

To assess the He absorption behaviors of the two phases (*γ* and *α*_2_) in the Ti‐48Al‐2W‐0.08B alloy, He ion implantations were performed at 750 °C. **Figure** **3** reveals the microstructural evolution of the nano‐lamellar *γ*/*α*_2_ under He irradiation—the Stopping and Range of Ions in Matter (SRIM) calculation results are provided in Figure S2 (Supporting Information). The *γ* and *α*_2_ phases show a significant difference in He gas bubble formation. A high density of He gas bubbles (≈2 × 10^22^ m^−3^) formed in the *α*_2_ phase, while the density of He gas bubbles in the *γ* phase is lower by a factor of ≈7, and there was no noticeable interfacial segregation of He bubbles. The He bubbles in both phases are faceted as shown in Figure 3a,b. No clear evidence of a bubble‐denuded zone was observed near the interfaces. The chemical mapping in Figure 3c–f suggests that no clear chemical segregation occurs near the bubbles or the interfaces. In the *α*_2_ phase, some features in white contrast form in the vicinity of faceted He gas bubbles. The high‐resolution TEM micrograph implies that irradiation‐induced planar faults form associated with the formation of He gas bubbles (Figure S3, Supporting Information).

### 2.3 Effect of He Embedding and Migration Energies on the Distribution of He Bubbles

The experimental results described above imply that the *α*_2_ phase may have a higher He absorbing capability. To understand this phenomenon, DFT calculations were performed. We first investigated the He migration energies, *Q*_m_. The minimum *Q*_m_ is found to be 0.31 eV for the *α*_2_ phase when the He atoms migrate along the crystallographic *c*‐axis. For the *γ* phase, the minimum *Q*_m_ is 0.43 eV when He atoms migrate along the *a*‐axis (or equivalently *b*‐axis). At the experimental ion‐radiation temperature of 750 °C (*T* = 1023 K), *Q*_m_ = 0.43 eV will lead to He interstitial lattice diffusivity as high as *D*_He_ = 10^−10^ m^2^ s^−1^. Since the characteristic diffusion distance scales as (2*D*_He_ *t*)^1/2^, this means >10 µm diffusion distance if just holding for *t* = 1 s, which is much greater than the *γ*‐*α*_2_ lamella spacing. These results indicate that “distance is no object” and He atoms anywhere can easily migrate to the nearest lower‐embedding energy phase (*α*_2_), explaining the higher density of He gas bubbles in the *α*_2_ phase observed in the experiment, while the *γ* phase shows much less He damage. Together with the calculations on $E_{\mathrm{emb}}$ below, this proves that the lower‐$E_{\mathrm{emb}}$ region can indeed shield/screen the higher‐$E_{\mathrm{emb}}$ region largely from He damage, even with ∆$E_{\mathrm{emb}}$ as small as 0.5 eV/He (see DFT calculation below). And if we are interested in protecting ferritic/martensitic steels, since $E_{\mathrm{emb}}$ in BCC Fe's grain boundaries is on the order of 4 eV/He (see Figure 1c), all we need to do for protecting such steels is to seek easy‐to‐disperse He‐absorbing nano‐phases with $E_{\mathrm{emb}}$ < 3 eV/He.

**Figure 4:**
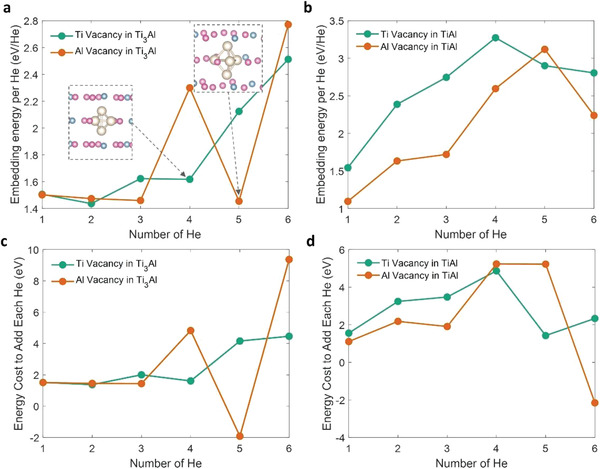
a,b) Embedding energy per He atom $E_{\mathrm{emb}}$ as a function of the number of He atoms *N*_He_ that are put into a single vacancy site for a) *α*_2_ phase and b) *γ* phase titanium aluminides. The green and red curves are for Ti and Al vacancies, respectively. Insets of (a) show the relaxed local structures of the He complexes for *N*_He_ = 4 in Ti vacancy and *N*_He_ = 5 in Al vacancy in the *α*_2_ phase, respectively. Purple: Ti; Blue: Al; Light beige: He. c,d) The marginal energy cost to add each He [*E*_emb_(*N*_He_) − *E*_emb_(*N*_He_ − 1)]. In (d), the marginal energy cost appears low when the number of He is >4, because the He escapes the vacancy site during the relaxation in DFT calculations.

Next, we illustrate that the He embedding energy $E_{\mathrm{emb}}$ strongly influences the He absorbing capability. We first considered pristine titanium aluminides without any defects. Single He atom was put in several inequivalent locations in the titanium aluminides and then the atomic structure was relaxed until the forces on each atom are lower than 5 × 10^−3^ eV/Å. The minimum embedding energies are found to be 2.89 and 3.36 eV/He for the *α*_2_ and *γ* phases, respectively. Thus, $E_{\mathrm{emb}}$ of the *α*_2_ phase is lower than that of the *γ* phase by 0.47 eV/He. Also, defects such as vacancies are inevitable in a lot of engineering materials. Hence we also investigated $E_{\mathrm{emb}}$ values for Ti and Al lattice vacancies in titanium aluminides, which provide a relatively spacious room for the He atoms and can potentially host them with lower energy costs. When a single He atom resides in their vacancy sites, $E_{\mathrm{emb}}$ is found to be ≈1.5 eV/He for both *α*_2_ and *γ* phase titanium aluminides, which is only half of the values for pristine *α*_2_ and *γ* phases without vacancies (**Figure** **4**).

The He absorbing capability may be further enhanced if a single vacancy site can trap multiple He atoms. To test this speculation, we put more He atoms in a single vacancy site and studied how the embedding energy per He atom $E_{\mathrm{emb}}\equiv E_{\mathrm{emb}}/N_{\mathrm{He}}$ varies with *N*_He_. The results are shown in Figure 4a,b. For the *γ* phase (Figure 4b), one can see that $E_{\mathrm{emb}}$ increases from ≈1.5 to ≈3 eV/He when *N*_He_ increases from one to four. Then $E_{\mathrm{emb}}$ stops increasing when more He atoms are put into the vacancy site in the initial structure. This is because some He atoms migrate to other sites spontaneously after the atomic structure relaxation in DFT calculations, indicating that a maximum of four He atoms may reside in a single vacancy site in *γ* phase TiAl. In contrast, the $E_{\mathrm{emb}}$ ‐*N*_He_ curve shows more interesting features for the *α*_2_ phase Ti_3_Al (Figure 4a)—$E_{\mathrm{emb}}$ drops significantly for certain *N*_He_, showing “magic numbers” features well known in cluster physics. For example, in the case of the Al vacancy, $E_{\mathrm{emb}}$ is below 1.5 eV/He when *N*_He_ = 5, while for *N*_He_ = 4 and 6, $E_{\mathrm{emb}}$ is higher than 2.3 eV/He. We examined the atomic structure with *N*_He_ = 5 and found that a triangular bipyramid He complex is formed. Such complexes are bound not by chemical bonds, but by van der Waals attractions, and have dissociation energies much weaker than those of chemically bonded species.^[^ ^19^ ^]^ Yet, the high‐symmetry van der Waals complexes can reduce the total energy, leading to a lower $E_{\mathrm{emb}}$. Similarly, in the case of *α*_2_ phase Ti_3_Al with Ti vacancy, a tetrahedral He complex forms when *N*_He_ = 4, which again reduces $E_{\mathrm{emb}}.$ This may explain the high He absorbing capability of the *α*_2_ phase Ti_3_Al observed in experiments, as compared with the *γ* phase TiAl where the optimal *N*_He_ is tightly one even with Ti‐ or Al‐site vacancies. These results also imply that He clusters in some phases can become metastable when the number of He atoms reaches certain values (e.g., 5); such metastability can in turn promote the formation of new clusters rather than the growth of existing clusters. The analysis above can be further verified by the trend of the marginal energy cost to add each He in the vacancy site [i.e., *E*_emb_(*N*_He_) − *E*_emb_(*N*_He_ − 1)]. One can see that the energy cost can be very small, and even turns negative when the additional He atom leads to the formation of the high‐symmetry He van der Waals complexes (Figure 4c).

### 2.4 High‐Throughput Screening of He‐Absorbing Nano‐Phases

**Figure 5:**
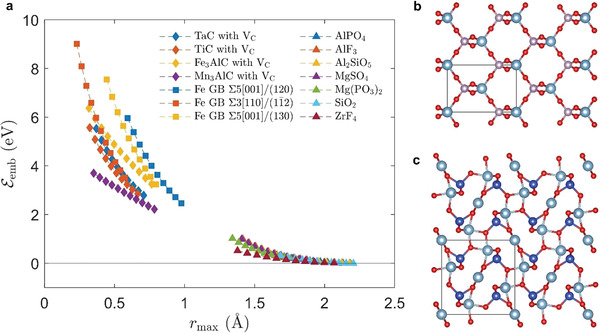
a) $E_{\mathrm{emb}}$ as a function of *r*_max_ for candidate materials in Table 1, carbides with carbon vacancy, and GB in BCC Fe. b,c) Crystal structures of b) AlPO_4_ and c) Al_2_O_3_·SiO_2_, which were identified to be candidate materials for He‐absorbing nano‐phases with large atomic‐scale free volume. The black box in (b, c) indicates the unit cell.

Combining insights from experiments and computations, one can see that atomic‐scale free volume, either with or without constitutional vacancies,^[^ ^16^ ^]^ can significantly decrease He embedding energy $E_{\mathrm{emb}}$. Given the matrix phase typically have either BCC or face‐centered cubic (FCC) structures with low free volume (see the left portion of **Figure** **5**a before the graph break), we expect that secondary phases are necessary to introduce such atomic‐scale free volume (see the right portion of Figure 5a after the graph break). Moreover, to prevent adverse effects on the fracture toughness, the size of these secondary phases *D* is desirable to be small (preferably *D* below ≈20 nm^[^ ^20^, ^21^ ^]^) as the oxide nanodispersions in ODS alloys. This size level would relieve the requirement for long‐range He migration as well. Here we carried out high‐throughput screenings for all compounds in the Materials Project database^[^ ^15^ ^]^ with Inorganic Crystal Structure Database (ICSD) numbers.^[^ ^22^ ^]^ In the following, we will discuss the screening process in sequential order.

#### 2.4.1 Neutron Absorption Cross‐Section

To ensure that He‐absorbing nano‐phases are neutron friendly, we consider only compounds consisting of elements with a thermal neutron absorption cross‐section below 1 barn, and a residual activity smaller than 10^5^ MBq kg^−1^ 10 years after 2 full‐power years of operation under DEMOnstration Power Plant (fusion DEMO) first wall and vacuum vessel radiation fields (see Figure 9 of Ref. [23]). Neutrons engendered as a product of burning plasma fusion need to reach a blanket, where they react with lithium‐6 to produce further tritium fuel for fusion reactions and heat up the coolant to drive a steam turbine and produce electricity.^[^ ^11^ ^]^ Therefore, the first wall and vacuum vessel, which are present in between neutron‐yielding plasma and the blanket, should have small neutron cross‐sections to allow the neutrons to stream through without significant absorption. This neutron cross‐section criterion leaves the following elements as adoptable constituents for He‐absorbing nano‐phases: aluminum (neutron absorption cross‐section *σ*_a_ = 0.232 barn), beryllium (0.0092 barn), bismuth (0.034 barn), calcium (0.43 barn), carbon (0.0035 barn), fluorine (0.0096 barn), hydrogen (0.3326 barn), lead (0.171 barn), magnesium (0.063 barn), oxygen (0.00019 barn), phosphorous (0.172 barn), rubidium (0.38 barn), silicon (0.171 barn), sodium (0.53 barn), sulfur (0.53 barn), and zirconium (0.184 barn). Helium, neon, polonium, cerium, and platinum also meet this criterion but got excluded, being either inert, radioactive, or expensive. Compounds consisting of other elements were excluded in this first stage of the screening process.

#### 2.4.2 Neutron Activity

Another important factor in designing fusion structural materials is neutron activity. Following the exposure to neutrons inside a fusion reactor, a certain amount of time is required for the material to decay to low‐level waste limits (< 4 MBq kg^−1^ for alpha radiation and < 12 MBq kg^−1^ for combined gamma and beta radiation).^[^ ^24^ ^]^ The elements with high neutron activation over long time horizons are thus undesirable from the nuclear‐waste‐disposal point of view. Here we used 10^2^ MBq kg^−1^ as an upper limit since the matrix material is likely to be either Fe or Ni, of which activities are ≈10^2^ MBq kg^−1^ 10 years after shutdown, assuming their use as a vacuum vessel component in a DEMO reactor for 2 years of full operation.^[^ ^24^ ^]^ This criterion then excludes cesium, cobalt, europium, lithium, samarium, and tellurium; in fact, these elements were already excluded by absorption cross‐section consideration, and thus, there will be no difference in the list of usable elements. One should note that long‐term activation should be considered for more thorough screening, but since the amount of the He‐absorbing nano‐phases would be on the order of 0.5 to 2 wt.% for acceptable mechanical properties, the activation by the added He‐absorbing nano‐phases would no longer be a problem provided that all the constituting elements have an activity < ≈10^2^ MBq kg^−1^, being reduced by two orders of magnitude.

#### 2.4.3 Elastic Moduli

For the next stage of the screening process, we exclude compounds with an average bulk modulus *B* smaller than 50 GPa or an average shear modulus *G* smaller than 20 GPa to ensure that the candidate materials are mechanically strong inclusions. The nano‐phases need to be capable of sustaining significant load imposed by absorbed He locally and resist dislocation cutting^[^ ^13^, ^25^ ^]^—after all, the difference between a second‐phase oxide in ODS and a gas‐filled cavity is that tensile stress can be transmitted across the former, while a cavity has zero moduli and sheds load on the surrounding metal. We can estimate this resistance by computing the second phase's elastic moduli. To that end, we utilized the dataset of elastic constants calculated from first‐principles DFT.^[^ ^26^ ^]^ For materials not included in Ref. [26], we used the statistical learning predictions of the elastic properties, which were developed in Ref. [27]. Our results indicate that despite the atomic‐scale free volume, many compounds still have large moduli and potentially good mechanical robustness.

#### 2.4.4 Free Volume

Finally, we considered the capability to absorb He. As discussed before, low He embedding energy is closely related to strong He absorbing capability. However, high‐throughput calculations of He embedding energy, which requires large supercells, can be computationally demanding. Hence, we resort to “easy‐to‐compute” metric. Based on previous analyses, we found that the size of the free volume to accommodate He atoms can be a tell‐tale signature that indicates low He embedding energy and large He absorbing capability. In this regard, we selected compounds with intermediate pore volumes in their crystal structures, which can potentially host He atoms with small embedding energies. We quantified the size of the free volume by the radius (*r*_max_) of the largest sphere that can be fitted in the crystal structure, without any atoms inside the sphere. Crystal structures used for the quantification are perfect lattices without any defects. One should also note that each atom in the host material is represented by a sphere whose radius is the covalent radius of the atoms instead of the ionic radius due to difficulties in taking into account its dependence on coordination numbers; we discuss later how well the *r*_max_ obtained using covalent radii correlates with the $E_{\mathrm{emb}}$. We then calculated *r*_max_ for all compounds that survived the previous two screening processes and kept only compounds with *r*_max_ > 1.5 Å. The *r*_max_ required to from a He tetrahedron is ≈1.7 Å when calculated using the van der Waals radius of He; however, since some atomic‐scale free volume can be elliptical, 1.5 Å is used for screening purpose. More stringent screening can be conducted by using a larger *r*_max_. The full list of potentially He‐absorbing nano‐phases obtained through these screening processes is provided in the Table S1 (Supporting Information), along with the estimated moduli and the size of the free volume.

#### 2.4.5 Melting Temperature, Phase Compatibility, and Wettability

When it comes to the processing and fabrication of a nanocomposite (by sintering, casting, or 3D printing^[^ ^28^ ^]^), the melting temperature, phase compatibility with the matrix, and wettability by the matrix material also become important for the selection of the nano‐phase. To be more specific, the fusion reactor vessel will be operating at temperatures up to 800 °C, and during manufacturing, the transient temperature could be even greater depending on the fabrication techniques used. The nano‐phase should thus be designed not to melt or dissolve into the metal matrix at the operating/manufacturing temperatures. Such phase compatibility can be checked by the CALculation of PHAse Diagrams (CALPHAD) method.^[^ ^29^ ^]^ Moreover, it is important to disperse nano‐phases uniformly throughout the matrix since aggregation can make their interface with the matrix have a longer end‐to‐end distance, which is detrimental from the stress amplification factor considerations (recall that oblate opening gives stress amplification factor →∞, while spherical and prolate do not when the end‐to‐end distances →∞). To enable uniform dispersion, the nano‐phases need to have a low enough wetting angle with the metal matrix. The wettability can be examined using the machine‐learning equilibrium wetting angle (*θ*) prediction^[^ ^30^ ^]^ method that we recently developed, which can predict arbitrary ceramic‐metal wetting angles. It should also be noted that diffusion may be available at such operating temperature, and hence, the nano‐phases could coarsen by the Gibbs‐Thompson effect.^[^ ^31^ ^]^ Fortunately, for many metal matrices, due to the low solubility of oxygen, nitrogen, etc., the nano‐phases are relatively stable (e.g., ODS steels) and coarsening‐resistant.

To demonstrate initial down‐selection of He‐absorbing nano‐phases with the above practical requirements, we took Fe as an exemplary matrix material. Since a method of predicting the melting temperature is available only for a limited range of ceramic materials,^[^ ^32^ ^]^ here we screened the candidate materials first using wetting angle values. Ideally, wetting angles should better be smaller than 90°, but since only a handful of compounds meet all the aforementioned requirements simultaneously, we relieved the wetting angle limit to 100°. We then considered melting temperatures and excluded compounds with a melting temperature lower than 800 °C and examined the remaining compounds’ phase compatibility with Fe via CALPHAD calculations using the Thermo‐Calc software with TCFE8 database. The weight fraction of the He‐absorbing phases was set to be 1 wt.%. For compounds with elements that are not included in the TCFE8 database, the phase compatibility was examined using the Materials Project database.^[^ ^15^ ^]^

**Table** **1** shows the resulting list of He‐absorbing nano‐phases for Fe. SiO_2_ was the only compound that survived the down‐selection processes among those that can be examined via CALPHAD calculations. Based on thermodynamic calculation (Figure S4, Supporting Information), the phase is predicted to be quartz. The crystal structure of the SiO_2_ that would appear at 800 °C is expected to be either P6_2_22 or P6_4_22 (mp‐6922 or mp‐10851 SiO_2_), which is called *β*‐quartz^[^ ^33^ ^]^ and is different from that of the mp‐546794 SiO_2_ in Table 1, I$\bar{4}$2d. The *r*_max_, the bulk modulus, and the shear modulus of the *β*‐quartz are estimated to be 1.40 Å, 109.8, and 67.8 GPa, respectively. The *r*_max_ is smaller than the criterion we used for screening, but He embedding energy is found to be very low (≈0.1 eV), as compared with that of the GBs of BCC Fe in Figure 1c. Therefore, we expect that SiO_2_ should be an attractive He‐absorbing nano‐phase for Fe‐based alloys. Such composites would better be prepared by low‐temperature manufacturing techniques that do not involve Fe melt since SiO_2_ is predicted to melt at ≈1450 °C when in contact with Fe, which is lower than the melting point of Fe (Figure S4, Supporting Information).

**Table 1:** List of Possible He‐Absorbing Nano‐Phases and their Compatibility with an Exemplary Matrix, Fe

Chemical formula	Material project ID	Avg. bulk modulus [GPa]	Avg. shear modulus [GPa]	*r*_max_ [Å]	Wetting angle [deg.]^a)^	Melting point [°C]	Phases at 800 °C for Fe with 1 wt.% of He‐absorbing nano‐phase
Al_2_SiO_5_	mp‐4753	155.8	97.5	1.90	81.8	1840	Fe + mullite [xAl_2_O_3_·ySiO_2_] + quartz [Si‐O] + spinel [Fe‐Al—O]
AlPO_4_	mp‐7848	84.6	48.2	1.92	90.2	1800	Fe + halite [Fe—Al—O]
SiO_2_	mp‐546794	93.8	57.5	1.93	95.1	1710	Fe + quartz [SiO_2_]
AlF_3_	mp‐468	116.1	53.0	1.64	89.1	1291	Fe + AlF_3_ ^b)^
Mg(PO_3_)_2_	mp‐18620	113.5	60.8	1.57	96.5	1160	Fe + halite [Fe—Mg—O]
MgSO_4_	mp‐4967	106.8	51.5	1.64	97.8	1124	Fe + halite [Fe—Mg—O] + pyrrhotite (Fe—S)
ZrF_4_	mp‐561384	133.4	54.7	1.73	97.2	910	Fe + ZrF_4_ ^b)^

^a)^ Wettability by molten Fe at 1600 °C;

^b)^ Equilibrium phases predicted by Materials Project database.^[^ ^15^ ^]^

On the other hand, AlPO_4_, Mg(PO_3_)_2_, and MgSO_4_ turned out to react with Fe and form other phases with phosphorus and sulfur elements being dissolved into the Fe matrix. Al_2_SiO_5_ would also react to form quartz and spinel phases, but it is still a viable option now that there remain mullite and quartz, which have large atomic‐scale free volume. Since fluorine is not included in the TCFE8 database, no detailed phase information is available for AlF_3_, and ZrF_4_; however, all these compounds are predicted to be thermodynamically stable when in contact with Fe at 0 K based on Materials Project database,^[^ ^15^ ^]^ having direct tie‐lines with BCC Fe (Figure S5, Supporting Information). It should be noted that the equilibrium phase and the corresponding moduli and *r*_max_ value could differ from those listed in Table 1 and that actual melting can take place at lower temperatures as the materials will be in contact with Fe.

### 2.5 Feasibility of the Identified Candidate Materials

To further verify that large *r*_max_ is an appropriate indicator of low $E_{\mathrm{emb}}$, we artificially strain the materials in Table 1, so that *r*_max_ can be modified continuously, then $E_{\mathrm{emb}}$ as a function of *r*_max_ is calculated using DFT calculations. The results are shown in Figure 5a. One can see that the *r*_max_ versus $E_{\mathrm{emb}}$ relationship almost collapses onto the same curve for candidates listed in Table 1. All of them showed $E_{\mathrm{emb}}$ far below that of the GBs of BCC iron (≳3eV/He ) and even smaller than that of most Y‐M‐O oxides (where M = Ti, Zr, Hf, Al; 0.59–3.14 eV/He^[^ ^34^ ^]^), which have been investigated extensively for the development of irradiation‐resistant alloys. The atomic structures of two exemplary compounds AlPO_4_ and Al_2_O_3_·SiO_2_ are illustrated in Figure 5b,c. The sizes of the atomic‐scale free volume are 1.9 Å for AlPO_4_ and 1.7 Å for Al_2_O_3_·SiO_2_. Using DFT calculations, the He embedding energies $E_{\mathrm{emb}}$ are found to be 0.08 and 0.10 eV/He for AlPO_4_ and Al_2_O_3_·SiO_2_, respectively, which are exceptionally low and far below that of the GBs of BCC iron, where the size of the free volume is found to be below 1 Å (the exact size is dependent on the angle and sigma value of the boundaries). This, combined with the experimental demonstration in Figure 3, gives us high confidence that these nano‐phases can soak up the He and protect the GB if they are distributed uniformly and close enough to the GB.

The possibility that He atoms and vacancies (V) self‐aggregate in the nano‐phase to form He bubbles needs to be assessed. Consider vacancy–helium complex V*_n_* He*_m_*. We distinguish between the situation of *n* = 0, *m*≥1 which covers the majority of the computational screening we have done where one relies on natural free volume in the crystal without any vacancy, with *n*≥1, *m*≥1 “He bubbles”. For fixed *n*, the existence of an optimal *m* that is *very small* could be advantageous in retarding coarsening. Figure 4c,d show the marginal energy cost of V*_n_* He*_m_*_‐1_ → V*_n_* He*_m_*, i.e., $\frac{\partial E}{\partial m}{|}_{n}\equiv \mu_{\mathrm{He}}$, the chemical potential of He. We see that *µ*_He_ is often minimized at *N*_He_ = *m* = 1. Then, the growth/coarsening of existing clusters in *m* would be less favorable compared to the formation of new clusters since He complexes with larger *N*_He_ become metastable. Also, the coalescence of multiple clusters V*_n_* He*_m_*, V*_n’_* He*_m’_*, inside the nano‐phase may be kinetically facilitated by the imbalance in the hydrostatic stress exerted by the different He clusters to break bonds in between. The suppression of the growth/coarsening due to a small optimal *m* would limit the hydrostatic stress and its imbalance, respectively. Hence, the evolution of He bubbles to the critical size would take a longer time. This may be the case in the very early stage of irradiation, where dpa is small (<1). However, under irradiation at high temperature, the size of the atomic‐scale free volume may grow via vacancy diffusion accelerated by radiation knock‐out, thermal activation, etc. without requiring deviatoric stress, facilitating the evolution of He bubbles. That said, if the evolved He bubbles remain inside a nano‐phase like in Figure 3b being “0D” openings, they are still less damaging as compared to He segregating at GBs, which are “2D.” The composite with such He‐absorbing nano‐phases would thus be more tolerant against embrittlement even when He bubbles eventually evolve in size.

There also remains the question of whether scalable fabrication of metal matrix composites with such nano‐phases is achievable, which has been a long‐standing hurdle in the industrialization of oxide‐dispersion‐strengthened alloys. In this regard, further exploration of advanced manufacturing methods is desired. For example, the formation of SiO_2_ nanodispersions by internal oxidation during selective laser melting was reported;^[^ ^35^ ^]^ this finding indicates that adding inoculants that can play a role as nucleation sites, thereby promoting the formation of nanoscale SiO_2_, could enable mass production. In addition, carbides and nitrides, which are typically more dispersable as compared to oxides, could also be a good compromise in the short run. If we loosen the neutron cross‐section constraint, more transition metal carbides/nitrides become available. In particular, compounds with empty substitutional sites (“constitutional vacancies”) such as interstitial carbides (e.g., TiC, VC) or *κ*‐carbides (Fe, Mn)_3_AlC could also be useful for He shielding. Early transition metals (i.e., group IV, V, and VI elements) have relatively large atomic radii and thereby have a carbon/metal atomic radii ratio smaller than 0.59.^[^ ^36^ ^]^ Carbides of these elements thus typically form a crystal structure where two FCC lattices are interpenetrating, which can also be viewed as an FCC array of transition metals with the carbon atoms inside the octahedral interstitial sites. These compounds often have large non‐stoichiometric ranges and can introduce constitutional carbon vacancies up to ≈10 at.%. Moreover, *κ*‐carbides, which have a perovskite‐type crystal structure, have inherent vacant sites in their perfect lattices.^[^ ^37^ ^]^ The He embedding energy of the vacancies in such compounds is relatively large as compared to those of the compounds identified by atomic‐scale free volume screening, as shown in Figure 5a. Nonetheless, it is still comparable to or smaller than that of the GBs of BCC iron, implying that the He atoms can be partitioned to the constitutional vacancies of these compounds rather than localizing onto the GBs. When coarsening resistance is considered, interstitial nitrides or *κ*‐nitrides (Fe, Mn)_3_AlN may also be of interest since the solubility of nitrogen in transition metals (e.g., Fe)—the primary constituent elements of the materials for structural components—is generally lower than that of carbon.

## 3 Discussions

In this paper, the effects of He embedding and migration energies in soaking up and shielding He damage were explored via He implantation experiment, post‐mortem TEM characterization, and DFT calculations. The results demonstrate that the incorporation of nano‐heterophases with ∆$E_{\mathrm{emb}}$ as small as 0.5 eV/He can guide He atoms where to reside at 750 °C. Also, calculations show that the optimal number of He atoms for a He cluster is discretized (“magic numbers” in cluster physics) and often quite small (e.g., *N*_He_ = 1 to 5), before the cluster would spontaneously break up. These details need to be accounted for when computing the exact He uptake capacity in designing secondary phases, some having few‐percent constitutional vacancies.

Based on these findings, high‐throughput screening to identify potentially compelling He‐absorbing nano‐phases was conducted using the following criteria: the compound should 1) consist of neutron‐friendly elements with a neutron absorption cross‐section and residual radioactivity smaller than 1 barn and 10^2^ MBq kg^−1^ ten years after 2‐year service, respectively; 2) have a bulk modulus and an average shear modulus >50 and 20 GPa, respectively, to be able to be load‐bearing; and 3) have atomic‐scale free volume with a radius >1.5 Å. Given the requirements, we have identified certain silicates, phosphates, and SiO_2_‐Al_2_O_3_‐based crystals and even glasses to be of great utility for soaking up He and shielding the matrix grain boundary network. Furthermore, compounds with few percent constitutional vacancies such as interstitial carbides/nitrides, *κ*‐carbides/nitrides, and yttria‐stabilized zirconia (YSZ)—8 mol.% YSZ having up to 4 at.% oxygen vacancies—may also shield GBs from He embrittlement. The nano‐phases identified in this work via the materials genomics search could help fusion structural materials become capable of soaking up a few‐thousand appm of He and shielding the matrix GBs from He damage—the main cause of their He embrittlement, while also providing superior high‐temperature creep resistance and dpa resistance like the ODS alloys.

## 4 Experimental Section

### Materials Fabrication, Irradiation, and Characterization

Ti‐48Al‐2W‐0.08B (at.%) alloys were fabricated using a spark plasma sintering process.^[^ ^38^ ^]^ A minimal amount of tungsten was added to improve mechanical properties. Two distinct microstructures were created by varying the processing conditions. Ti‐48Al‐2W‐0.08B alloy with semi‐coherent nano‐lamellar *γ*/*α*_2_ structures was fabricated under 50 MPa at 1375 °C. The measured temperatures were 60 °C below the actual sample temperatures due to the thermal gradient between the sample and the location where the temperatures were measured. He ion implantation was performed at 750 °C to a fluence of 1.6 × 10^16^ cm^−2^ using He ions with the energy of 800 keV. The He concentration was calculated to be ≈10000 atomic‐parts‐per‐million (appm) at the penetration depth of ≈2.25 µm beneath the surface by the Stopping and Range of Ions in Matter (SRIM) code with Kinchin‐Pease approximation^[^ ^39^, ^40^ ^]^ (Figure S2, Supporting Information). The microstructure of Ti‐48Al‐2W‐0.08B alloys was characterized by LYRA 3 TESCAN SEM and FEI Titan TEM. A TEM‐based EDS imaging was used to probe the chemical distribution in the as‐fabricated and irradiated specimens.

### Density Functional Theory Calculations

The Vienna ab initio simulation package (VASP)^[^ ^41^ ^]^ was used to do the ab initio calculations based on density functional theory (DFT).^[^ ^42^, ^43^ ^]^ The exchange‐correlation interactions were treated by generalized gradient approximation (GGA) in the form of Perdew‐Burke‐Ernzerhof (PBE).^[^ ^44^ ^]^ The core and valence electrons were treated by projector augmented wave (PAW) method^[^ ^45^ ^]^ and plane‐wave basis functions, respectively. Supercells with a length >10 Å along each dimension were used to calculate the He embedding energies, and the first Brillouin zone was sampled by a 3 × 3 × 3 ***k***‐mesh. The atomic structure was relaxed until the force on each atom was <5 × 10^−3^ eV/Å.

### Thermodynamic Calculations

Equilibrium phases were identified using the CALculation of PHAse Diagrams (CALPHAD) technique in conjunction with Thermo‐Calc software. TCFE8 databases for Fe‐based alloys were used. The calculations were carried out for compositions corresponding to Fe with 1 wt.% of He‐absorbing nano‐phases.

### High‐Throughput Materials Screening

The Python Materials Genomics (pymatgen) package was used to retrieve information (compositions, atomic structures, etc.) on all compounds in the Materials Project database,^[^ ^15^ ^]^ and the Atomic Simulation Environment (ASE) package^[^ ^46^ ^]^ was used to analyze the atomic structures.

## Conflict of Interest

The authors declare no conflict of interest.

## Author Contributions

H.X. and S.Y.K. contributed equally to this work. H.X., S.Y.K., C.S., and J.L. designed the research inspired. J.‐P.M. and T.V. prepared samples. D.C. carried out He ion implantation. C.S. performed transmission electron microscopy analysis. H.X. conducted density functional theory calculations. S.Y.K. conducted thermodynamic calculations. H.X. and S.Y.K. analyzed the results and worked on high‐throughput screening. H.X., S.Y.K., C.S., and J.L. wrote the paper. All authors contributed to the discussion of the results and commented on the manuscript.

## Supporting information

Supporting Information

## Data Availability

The data that support the findings of this study are available from the corresponding author upon reasonable request.
