# Supplementary material for: Materials Genomics Search for Possible Helium‐Absorbing Nano‐Phases in Fusion Structural Materials
Source: Adv Sci (Weinh). 2022 Sep 30;9(32):2203555. doi: 10.1002/advs.202203555 (PMC9661827; doi:10.1002/advs.202203555)
Supplement: Supplementary file 1 — Supporting Information [file ADVS-9-2203555-s001.pdf]

Materials Genomics Search for Possible Helium-Absorbing Nano-Phases in Fusion Structural Materials

Haowei Xu[1,†], So Yeon Kim[2,†], Di Chen[3], Jean-Phillippe Monchoux[4], Thomas Voisin[5], Cheng Sun[6,*], and Ju Li[1,2,*]

[1]Department of Nuclear Science and Engineering, Massachusetts Institute of Technology, Cambridge, MA 02139, USA

[2]Department of Materials Science and Engineering, Massachusetts Institute of Technology, Cambridge, MA 02139, USA

[3]Department of Physics and Texas Center for Superconductivity, University of Houston, Houston, TX 77204, USA

[4]Centre for Materials Elaboration and Structural Studies, University of Toulouse, French National Centre for Scientific Research, Toulouse 31055, France

[5]Materials Science Division, Lawrence Livermore National Laboratory, Livermore, CA 94550, USA.

[6]Characterization and Advanced PIE Division, Idaho National Laboratory, Idaho Falls, ID 83415, USA

[*]Corresponding authors: cheng.sun@inl.gov (C. Sun), liju@mit.edu (J. Li)

[†]These authors contributed equally.

Supplementary Information

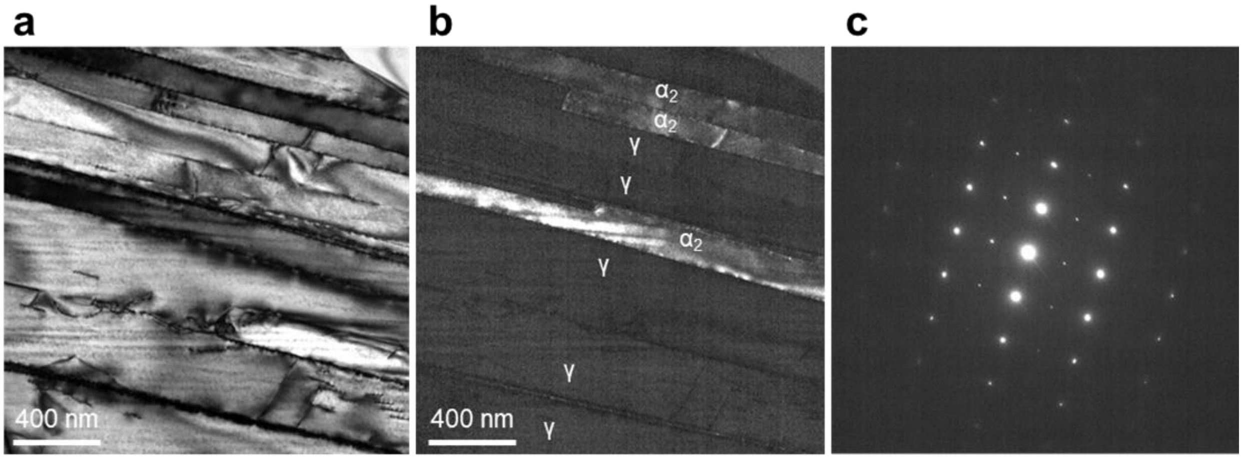

Figure S1. Microstructure of Ti-48Al-2W-0.08B (at.%) alloy before He irradiation. (a) Bright-field image showing nano-lamella structure. (b) Dark-field image highlighting α_2 phase. (c) Corresponding selected area diffraction pattern (SADP).

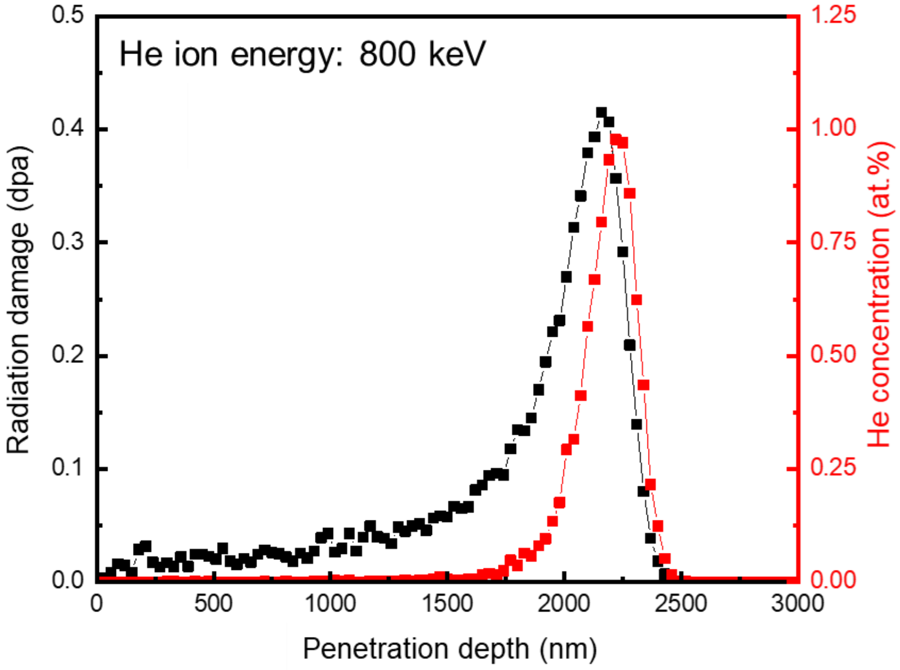

Figure S2. Stopping and Range of Ions in Matter (SRIM) calculation results. The energy of the He ion used is 800 keV.

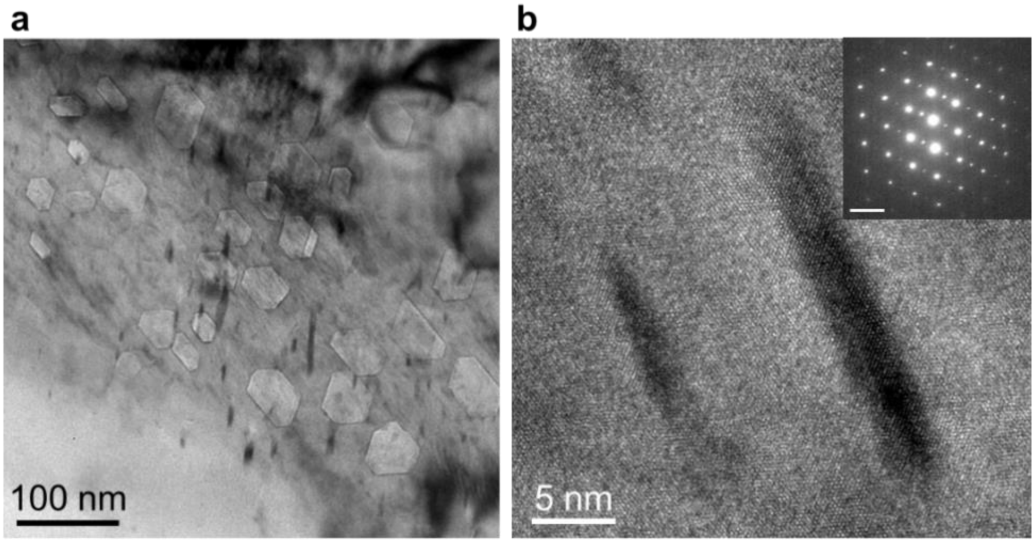

Figure S3. α_2 phase in Ti-48Al-2W-0.08B alloy after He irradiation. (a) Bright-field image showing faceted He bubbles and planar faults. (b) High-resolution TEM (HRTEM) image of planar faults showing lattice fringes. The inset displays the corresponding SADP. Scale bar in the inset, 5 nm^{-1}.

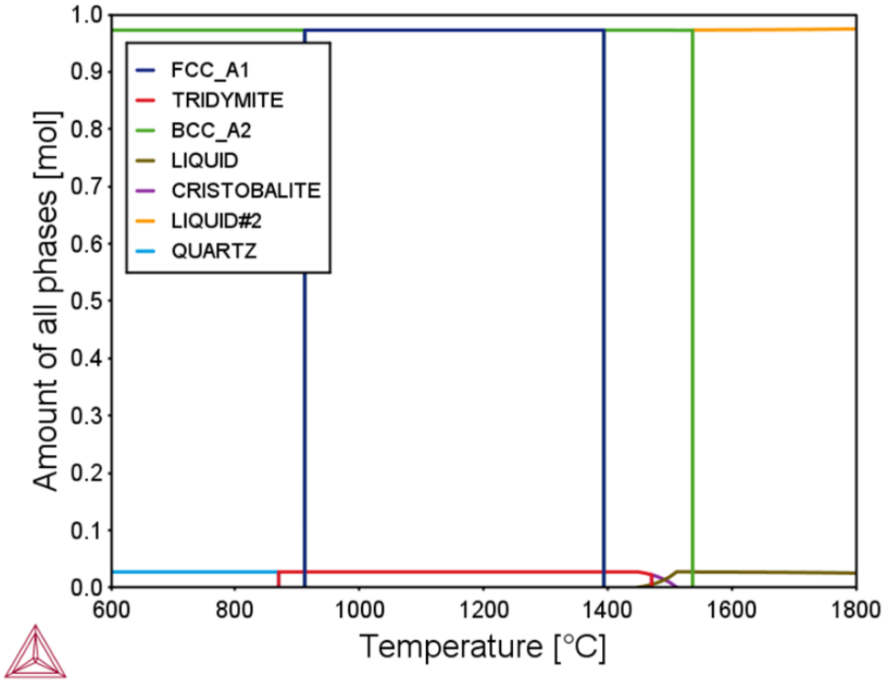

Figure S4. CALPHAD calculation results for Fe-1wt.% SiO_2.

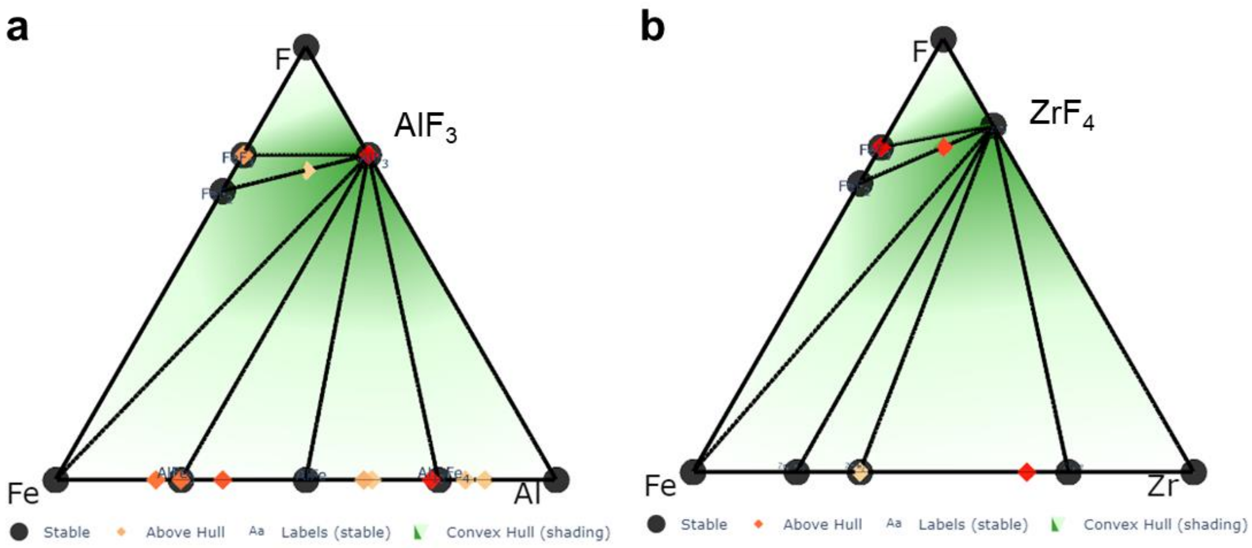

Figure S5. Ternary phase diagrams at 0K predicted by Materials Project database[15].

Table S1. List of candidate materials for He-absorbing nano-phases.

Formula	Materials Project ID	r_{max} (Å)	Average Bulk Modulus (GPa)	Average Shear Modulus (GPa)
Ca72P48O192	1197377	1.7405	91.7124	57.6904
P24Pb12O84	1203776	1.624	87.4954	39.5014
Mg10C8O36	1204170	2.2139	79.835	40.8148
O144Al48Ca72	12147	1.63	96.5546	64.649
Ca2H8S2O12	23690	1.6223	80.7394	40.6891
Bi2P8H2O24	24348	1.8146	92.2017	43.3545
Zr4P4O18	27132	1.6141	132.3133	71.1828
Bi4P12O36	27135	1.6953	98.1256	47.8368
P8H32O36	27141	1.6358	83.9183	43.67
Sn12P8O32	27493	1.9227	72.8556	31.9319
Na8Si4H64O44	504605	1.7109	74.2028	44.5514
Al6P18O54	540549	1.6313	117.7959	65.6274
Na8H40S8O32	540554	1.6875	58.5082	31.7716
Zr4P8H16O36	540737	1.8927	85.4084	39.6502
Na4P4H16O20	554288	1.504	72.8155	41.3985
H8C4O8	558412	1.6157	73.8663	43.2691
Si24O48	640556	2.0947	96.3286	59.1507
Na2Zr4P6O24	6475	1.6376	101.496	58.5587
Ca8Al16Si16O64	6532	1.6502	114.7239	73.7317
Sn4P4H4O16	690702	1.5619	90.7471	37.288
Na8P4H8C2O18	696155	1.6816	68.8678	39.6117
Na4P4H24O24	696157	1.6132	72.0756	41.5278
Ca2P4H12O18	706278	1.9702	84.0596	43.783
Zr2H12O6F8	706386	1.6191	91.4083	41.9525
Ca4P4H8O18	706426	1.9358	89.7576	47.1621
H20C4S4O20F12	706514	1.9909	62.303	28.2247
Na4P2H22O16	720570	1.587	64.6977	38.1182
Na6P6H24O30	720825	2.0493	66.7233	37.7363
Mg10H20C8O36	720835	2.0146	100.7309	56.2109
Al8H72S4O60	720965	1.9613	64.308	33.0917
Na8H16S8O32	721314	1.7593	63.9215	32.1772
P3Pb2O10	1104631	1.5581	97.7423	44.3639
C2F8	1167	1.578	56.7312	24.3128
Sn4P4O16	1179348	1.6542	91.0928	40.9242
CaAl6H12S4O28	1194752	2.2092	114.0377	56.2643
H32C20	1195106	2.0808	88.8909	70.0902
MgH30C2S2O18	1195604	1.9088	62.1897	34.607
Rb4C12S8O16F24	1196669	1.8808	51.9932	21.8926
Ca2Al4Si6O26	1196952	1.9503	84.8073	45.5
Mg4C4O24	1198523	1.6728	70.6594	34.8836
Mg2H44S2O30	1198750	1.7346	72.4319	39.1611
Na2Mg8Si12O36	1199476	2.1299	107.777	64.9519
Ca4Be4P4O20	1200025	1.5752	111.3424	68.6333

Na4Al12P8O56	1200599	1.605	91.3113	45.1959
Ca2Al4Si6O26	1202125	1.9555	86.4554	47.7778
Ca2Al4Si6O26	1202493	1.8978	85.7494	46.1504
Ca18Mg2P14O56	1203362	1.539	101.5504	62.9555
Na8Mg8Si24O60	12187	1.8457	100.0787	65.9129
Si4P8O28	18157	1.6398	125.8912	71.2406
Mg4P8O24	18620	1.5735	113.4945	60.8447
Mg8P8H56O56	23734	1.5628	95.6193	51.4844
Mg2H4S2O10	24041	1.5164	96.3966	44.7655
H20S2O16	24118	2.0224	71.5706	36.9579
Mg4P8H64O40	24195	2.2209	70.6534	38.1195
H12C4S4O16F12	24357	1.8668	62.9417	27.444
Ca2P4H8O16	24361	1.6315	95.1867	48.6389
Al4P4H16O24	24398	1.8887	112.7406	58.8558
Mg2H20S2O18	24399	1.9075	78.6975	41.0565
P8H32O32	27278	1.7888	73.1836	38.1735
Al8H24O24	27540	1.5166	121.956	67.8235
Ca4P16O44	30983	2.0608	80.0828	42.2223
Si5P6O25	3273	1.8666	114.6188	65.9375
Na4H36O20	505077	1.5184	66.9469	39.4236
Ca6Si2H60C2S2O50	541025	1.6896	81.3038	46.5009
Sn4S4O16	542967	1.5858	76.3099	32.7471
H24C48	603334	1.9289	108.6158	87.7048
Ca4Be8P8O32	6772	1.573	123.3375	78.8957
Ca2P4H8O16	706585	1.5134	91.4182	46.0211
Ca2Al4Si8H8O28	706640	1.9224	81.5761	42.8051
Na20P20H72O56	706715	2.0815	55.467	30.8115
Na4P4H28O26	720334	1.5328	73.3842	42.3224
H36C4S4O28F12	720860	1.7735	67.9568	32.3504
Na8Zr4Si24O72	1180470	1.7827	89.5155	54.1536
P4Pb2O16	1191461	1.6899	67.0656	29.381
Al2P4O22	1193181	1.762	61.2627	27.2905
Ca2Mg5Si8H2O24	1196550	1.95	124.0886	72.901
Zr4P8O36	1197919	1.658	84.495	40.3143
Al8Pb4C8O44	1198485	1.6712	88.7873	42.7131
SiF4	1818	1.6869	56.6274	24.9663
MgPbF6	19734	1.5215	82.1136	23.3038
Na4Al4H96S8O80	24046	1.7253	71.8991	39.2402
Na4Si2H24O18	24369	1.5182	76.2931	46.1902
Na5Zr2F13	27391	1.8898	61.7106	29.4138
Ca12Be17O29	27407	2.3728	103.1366	76.083
Zr4P8O28	5024	1.6378	100.3762	52.9179
Be3F6	558118	1.6331	85.205	45.8618
Mg6P4O16	560623	1.5456	124.689	70.0135
Si4O8	6945	1.8405	97.3637	59.8221
Mg3P2H44O30	706658	1.7357	83.6087	46.1854
Ca4Al4Si12Sn2O44	867974	1.7658	95.4726	52.496

Na4P4O16	1180166	1.7764	56.7053	29.9807
Al2O6	1182877	1.7642	67.9401	30.8835
Na2Al2C2O10	1189078	1.5385	73.6401	45.7475
Ca2P4O18	1190799	2.2043	56.152	25.3889
Zr2S4O18	1191549	2.2676	77.6418	34.7197
Al4Si4O18	1192644	1.9455	96.6292	51.7815
Zr4S8O36	1196466	1.9942	75.6914	33.7138
Al4P4O24	1202156	1.8682	74.532	35.2822
Al8C2O8	13703	1.5843	126.0307	79.1245
C4O8	20066	1.7609	72.8724	45.1939
P8O20	2173	1.6223	98.5295	52.9268
Si12Pb18O42	21767	1.6243	94.1763	40.2286
Mg3Si2H4O9	23764	1.8906	109.3451	58.4572
P4H12O16	23902	1.6757	77.3302	39.5851
H24O12	24043	2.3089	80.058	44.1419
Ca2P2H10O12	24389	1.5388	89.475	48.604
Mg4H56S4O44	24396	1.9113	78.0302	41.49
Mg4H32S4O32	24397	1.5298	86.2941	44.4406
SnF4	2706	1.5166	71.3119	22.8537
Na2Al22O34	3405	1.6093	146.286	96.1062
P16Pb8O48	3476	1.9234	92.0227	43.5359
Al2P2O8	4051	1.8253	87.3034	49.8163
Si4P8O28	4769	1.7487	123.3469	69.8028
BeSO4	5046	1.6057	95.7764	52.3529
Mg4H48S8O36	504802	1.6015	71.0734	36.4962
P16O28	542622	1.7649	60.0739	31.5711
P20C20F60	556383	1.6832	50.5448	21.6603
Na2Ca2C3O9	557391	1.636	90.6593	65.2573
P8O20	562613	1.8675	65.8985	35.5204
Ca2P2H8O12	632758	1.5255	80.5624	41.5656
Na8P4H40O34	721144	1.8722	51.9996	28.3698
Si4O8	7648	2.0879	90.1345	55.1187
Al2Si2O9	1103547	1.9424	96.5753	51.7942
Ca2Mg5Si8O24	1129406	1.9463	120.9335	73.9789
Be2H16S2O16	1183513	1.7546	79.4364	42.6745
Na2Zr2Si4O14	1191704	1.6122	99.2869	62.163
Na4Al4Si6O24	1196695	1.8129	75.4796	46.2551
Ca12Si12O38	1200566	1.9415	91.0726	58.5859
Ca2Al4Si8O28	1201693	2.5039	76.6161	42.2929
Ca7Si16O40	1203093	3.1607	87.8394	54.815
Be2H16S2O16	23996	1.7714	76.3242	41.0955
Ca2Mg5Si8O22F2	557662	1.8914	125.4838	77.0622
Na8Si6SnO18	560597	1.7537	90.5157	61.7033
Rb8H40C56S8O40	604583	2.0492	67.745	44.3413
Ca4Be4P4O16F4	6899	1.5782	117.8448	72.6974
Mg3Si4H2O12	696497	1.7889	124.3398	68.3046
Al2H24O10F10	721369	1.8749	59.2359	28.0167

Sn2P2O6	1095139	1.7638	60.1895	25.7324
Mg3Si2O9	1103891	2.1736	91.2407	47.46
Be6Al4Si12O38	1195857	1.6836	132.3668	82.7268
Mg12Si8O36	1197475	2.1765	90.462	46.7457
Ca2Al4Si8O28	1197555	2.4326	76.7883	42.5537
Zr4S6O36	1204554	1.7214	72.5601	29.9944
H16O4F8	23798	1.7321	73.216	33.8353
H12O4F4	27714	1.7483	76.9015	37.4142
Si12P12	2798	1.7906	51.6748	30.9343
Ca3S3O12	3082	1.9213	76.9353	41.0379
P8O12	368	2.002	51.7771	26.7607
Al8Si4O20	4753	1.9014	155.8419	97.4775
Ca18Mg18P24O96	646967	1.5018	104.7671	62.8292
AlPO4	7848	1.9175	84.6184	48.2478
Si2O4	7905	1.7765	101.2629	59.2832
Si8O16	8059	2.062	92.8801	56.9072
Na4P6H2O18	978284	1.6038	82.1261	46.1643
Al2Si2O11	1103571	2.2924	61.2004	29.5619
Mg3Si2O9	1104479	2.0938	92.3723	47.7884
Bi3P3O12	1105857	2.1049	86.9657	37.8047
Na4Ca2P4O22	1180413	1.5164	58.4334	29.3517
Zr4O4F16	1190734	1.8128	79.6049	27.2829
Al4Si8O24	1196937	1.9537	117.5366	69.5446
Ca2Al4Si12O40	1197130	2.1858	81.0404	43.5613
Sn8P4O20	1198177	1.5343	86.3399	36.2181
Mg6Si8O24	1200396	2.0314	113.9336	64.9806
Mg6Si8O24	1200846	3.798	65.2446	36.5393
Ca2Al4Si12O40	1201995	2.2063	83.3128	45.8908
Ca2Zr2Si12O34	1204163	2.2705	96.1424	52.9014
Al4O12	1205393	1.7372	71.1095	31.3458
Al8P24O72	14011	1.9129	104.7475	58.4075
Si12Pb12O36	21723	1.6605	105.0196	46.8449
Si8H8O12	24431	1.8221	68.2202	35.9333
Na8Si8O20	3193	1.5037	81.9662	58.2366
Be12Si6O24	3347	1.6925	172.9872	124.2732
Mg4S4O16	4967	1.6353	106.8052	51.5445
Na12Mg4C8S2O32	540866	1.5507	80.9651	55.8294
P16O36	541846	1.6859	78.2582	41.8923
Al8Si4O20	640357	1.7372	146.2113	87.6884
Ca20Si8C4O44	6796	1.627	102.6791	70.2945
H14O8	696717	1.554	74.9912	35.7062
H24O12	703459	2.2038	58.5063	33.1799
Si4O10	867933	1.7964	84.3047	47.4625
Pb2CO6	1079073	2.1689	55.1906	20.9892
H	1096977	1.6023	63.3231	37.5205
Al3F9	1103295	1.8693	109.756	49.9725
Al2Si4O12	1105328	1.6492	122.951	73.4466

Na4Al4Si6H8O24	1173798	1.7831	82.2831	48.8343
Ca2O20	1182183	1.5933	57.0129	21.825
Ca6Al4S2O16F16	1198254	1.6744	76.5095	34.0133
Mg28Si32O96	1200145	1.8423	127.1848	74.6615
Ca6Al4S2O32	1203850	1.6222	64.3917	27.6621
Mg6P4O16	14396	1.5545	107.9513	60.5199
Sn4P8O28	17887	1.5942	89.2282	44.1776
Ca6Al4H12S2O16F16	23872	1.65	91.4975	42.2419
H12O2F8	28003	1.701	66.2254	29.3649
Al4S6O24	4417	1.9584	102.0359	52.5038
C4	48	1.5537	155.3233	147.1173
Na4Al4Si6H8O24	510599	1.9196	66.7252	36.2651
Si4O8	554573	2.1392	93.2809	57.1902
Si4O8	559091	2.2537	89.2873	54.5924
C4	569304	3.6001	99.9763	92.1904
C8	569416	3.6057	135.2649	126.8414
Be6Al4Si12O36	6030	2.8273	131.2948	84.6128
C12	606949	3.7288	116.3205	108.2134
H8O4	634812	1.822	77.1726	42.2462
H24O12	696735	1.6123	74.7972	41.5004
Na2Mg2P2H24O22	703517	1.8184	68.9161	37.6602
Al2Si4O11	707135	1.7489	116.4063	71.2194
Ca2Al4Si6H12O26	707746	2.0472	93.7148	51.5392
Al4Si4H8O18	721216	1.89	119.8868	65.149
AlSi4O10	1105075	1.6842	101.2529	60.3623
Al8O24	1182886	1.7429	70.5726	31.0821
Na8Be4Si12Sn2O40	1196126	1.5208	92.568	57.9064
Be4Al4Si4O20	1196267	1.5218	154.8739	96.3383
Na16H112C8O80	1198167	1.7818	68.0519	41.5136
Al8Si16O48	1199589	2.8022	67.9298	39.0204
Na12P16O48	1200977	1.6847	77.4914	46.1208
Na12H4S8O32	23780	1.8699	64.4309	34.046
Ca2P4H12O14	24452	1.6548	71.4904	37.3648
H12S4O16F4	24463	1.5227	65.7716	29.1291
Mg4H16S8O36	24464	2.3982	62.1394	27.8009
Sn16S8O40	28025	1.7961	65.6576	26.5365
P8H24O24	28157	1.7204	67.7957	34.8543
Al2F6	468	1.6436	116.1179	52.9938
CaSnF6	4782	1.8704	60.5553	20.5271
Mg14Si16O44F4	559177	1.9419	129.2081	75.9841
Si12O24	559872	1.5707	109.4257	67.5841
Zr8F32	561384	1.7255	133.4394	54.7338
Ca12Si12O36	649676	1.7652	62.374	38.3568
Na10P6H24O32	703521	1.6039	67.4788	38.8233
H16S16O56	707293	1.9903	54.0787	23.5377
Na16Sn16H32O40	707767	1.5101	56.8999	23.5167
Al4P8H20O36	721876	1.6688	106.1023	54.0368

Na2Al2Si6O16	721988	1.5954	103.4997	69.043
Na2Ca8Si16H32O56F2	722188	1.5441	90.2642	50.3565
Zr2P4S12	8203	1.9567	51.4021	23.6975
Na4Al4Si4O16	8351	1.571	82.6365	57.5505
Si2O4	8352	2.1207	81.7113	49.6754
Rb2SiF6	10492	1.529	54.4364	23.0006
Si8Pb8O16F48	1179522	1.5569	68.0483	22.9896
Na4Zr4P8O36	1180130	1.9354	81.2613	44.2928
Ca3S6O30	1182310	2.0558	56.0417	23.4646
Al2O6	1182868	1.7404	81.2689	35.9802
Na2H4S4O8	1190311	1.6421	53.8165	24.641
Si96O192	1195265	4.1334	70.8132	42.7239
Na16Zr4Si24O72	1198341	1.6503	85.2297	53.9898
Al8H128C48O112	1201838	2.2924	87.5088	51.8461
Mg2H12S8O32	24460	1.71	73.3759	33.1735
Al20Si4O38	28503	2.055	152.4299	94.4829
Si48O96	644923	4.2039	68.6832	41.3753
Si28O56	651707	3.7631	77.2571	46.832
H4S2O8	690733	1.7087	66.9955	30.5289
Si2H28O8F12	707839	1.8206	69.7835	33.2853
Ca6Be4P8H20O40	707876	1.5982	99.1214	54.1893
Si4O8	7087	2.3381	81.7334	49.6931
Si2H20O8F12	722246	1.6432	77.7807	36.5027
P8O16	562065	1.7128	70.8165	37.6434
Al8H80S8O72F8	730446	1.8654	85.5796	43.8723
Na4P4H24O22F4	1176368	1.7421	63.5794	34.761
C122O4	1197923	2.7866	140.7724	127.0612
Ca8C16O40	1199283	1.6299	82.5152	50.6186
Sn4P8Pb4O32	22821	1.7498	104.6407	47.5974
MgH12SO9	23887	1.5673	79.4934	42.1242
Sn8P4C4O24	559291	1.6441	83.4878	39.2632
Mg2F4	560236	1.8393	54.8201	22.9535
Si34O68	561090	4.2323	68.2381	41.0744
H8O4	697111	2.2452	54.5916	31.1199
Na4Si2H20O16	707840	1.722	68.8702	41.965
Si34O68	8602	4.6837	67.2889	40.4937
Si8O16	556961	2.0176	88.839	54.2981
Al3P3O12	562607	1.7123	59.3482	23.568
Mg12P8O48	867981	2.1449	69.6437	30.6249
ZrS2	1186	1.5708	66.9983	37.4661
C28	1194362	2.2285	67.6256	55.9158
Ca8C16O32	1196658	1.598	68.8933	43.4868
Na4Si16O52	1202598	1.6077	69.2266	34.1974
C48	1203645	2.1371	88.9512	77.0279
Si34	16220	2.4562	52.9905	34.4654
H68C38	30168	1.9826	79.3128	62.6819
H16C28O4	30184	1.7734	92.7948	69.2881

Si4O8	555891	2.1719	86.0405	52.4806
Si16O32	556469	4.5561	67.76	40.7873
Si18O36	560973	3.5199	72.3131	43.5973
C60	630227	2.729	162.6578	150.0637
Si32O64	646895	4.6096	67.5499	40.6428
H88C40O12	707412	2.3605	56.9447	39.5991
H88C40O12	707910	2.1969	81.8107	59.173
Si4O8	972808	2.0148	95.385	58.5281
Na4Zr4P8O32	1197487	1.7446	85.7712	48.8065
Ca2H24S4O18	1198182	1.7894	73.1105	38.3492
P4H8Pb2O8	24298	1.6953	67.9315	26.7519
H36S2O24	31373	1.8512	67.2504	35.939
H30S2O21	31374	1.9904	77.5645	40.6634
Ca8P16O48	5680	1.5792	100.9928	57.0989
Be4P8H16O16	695815	1.6364	65.8538	34.8353
Mg2P4H24O22	722397	1.7984	78.8166	42.1269
H2	1066989	1.5774	54.2713	34.4667
Zr4O12F20	1199395	1.8739	67.6735	23.6341
Na4Ca4Al12Si18H32O76	1204300	2.0484	89.3378	50.4148
Si12O24	16964	3.3842	79.2709	48.1395
P8Pb6O26	18022	1.6003	96.9231	44.0381
MgAl2P2H18O18	24505	1.5509	104.8335	56.9203
Bi4P8O26	28541	2.2178	94.4156	44.2939
Na8P8H16O32	504944	1.6539	68.0462	37.2636
Al4Si4H8O18	541152	1.8515	120.707	65.5923
Si12O24	556218	3.4069	77.2707	46.8582
Al10P10O40	557842	3.6031	65.1973	36.8115
Si24O48	557933	3.849	79.3358	48.1811
Si24O48	560809	3.9514	75.1548	45.4961
Si12O24	560826	3.4572	74.2439	44.907
P24O24F36	565711	1.8646	52.9915	23.49
Si14O28	615993	3.6828	72.0654	43.5199
P4H12O16	626449	1.6625	84.7043	43.1882
P8H8O8F16	698060	1.7816	54.8862	21.8319
Ca8H16S16O64	703574	1.8357	82.7807	36.9038
Al16P16H32O88	706302	2.1966	78.9434	38.9238
Al4H44S4O40	722527	1.9174	83.2801	44.0995
P2F10	8511	1.807	55.4548	21.905
Na4Zr2P2C2O20	1180616	1.5742	76.8472	44.4282
Zr2H12O4F12	1192835	1.5759	88.1272	38.2472
C240	1196583	3.0059	133.707	122.4639
H4C4S4O12F12	1201933	1.7622	57.1507	24.3157
Si34C2O68	1204567	3.2262	68.6125	40.9876
Si6O12	12787	1.5177	113.5028	70.2659
Si18O36	15078	3.6992	70.3275	42.4164
Rb6C60	16975	2.803	111.614	111.0292
Zr6S2O18	28620	2.1756	138.9024	70.9187

C120S32	28709	2.7911	78.9554	61.7914
Sn4S8O24F8	554599	1.5717	60.0393	25.3822
Rb2NaAl6F21	560570	1.8657	80.4773	38.6647
Al8P8O44F4	653569	2.0229	72.548	34.3204
Si56O112	653763	4.0718	67.3119	40.4783
Ca6H6S6O27	698074	1.603	88.2156	42.1368
Al6P6O24	707977	4.0933	55.1623	30.9816
Al16F48	1323	1.7768	108.1198	49.1237
Na2Sn4P6O24	22163	1.7221	99.9356	47.3502
P16S4O24	28673	1.8533	51.1515	26.111
Al16P16O64	554273	2.5831	73.5785	41.7008
Al6P6O24	557604	3.5467	72.4804	41.071
Rb2Be4P6O20	557673	1.5122	88.7546	54.9188
Ca8Bi16O32	558751	1.531	84.6046	27.1062
Al16P16O64	561027	2.7072	65.3014	36.87
Na2Sn4P6O24	6525	1.6133	101.174	47.962
Mg4H40C4O32	695987	1.8726	87.0258	49.6924
Ca6H6S6O27	696075	1.6054	84.8014	39.9962
Mg8P8H48O52	721158	1.7473	95.5416	50.9976
Ca7Si6H4CO23	733443	1.5398	101.154	60.0921
Na8P8H12O30	740750	2.1601	67.2477	36.6244
Si20O30	1179195	2.1021	59.7846	32.384
MgPH13O9	1190717	1.5754	81.8903	45.0039
Al4P4O20	1193321	2.0204	88.4554	46.0728
Al8Pb12F48	14941	1.5261	95.5383	31.598
Al4P6H6O18	23995	1.9457	100.6911	50.4539
Mg6Si4H8O18	24097	1.9076	108.8159	58.1717
Si2O4	546794	1.9257	93.792	57.5299
Si4O8	554089	1.823	99.0004	60.8857
Si4O8	554151	2.0928	89.9877	55.0393
Si4O8	555147	1.6711	118.3236	72.7059
Si4O8	555251	2.655	77.3503	46.895
Si4O8	556319	2.0239	103.6979	63.0214
Si4O8	556376	1.7303	123.634	72.799
Si4O8	556553	1.7367	114.5112	70.2153
Si4O8	556588	1.6527	144.2407	86.924
Si4O8	556662	2.0006	101.5374	62.0276
Si4O8	556788	1.501	127.0031	76.7715
Si4O8	556880	1.8832	99.757	60.3336
Si4O8	556985	2.5335	82.1959	49.8037
Si4O8	556994	2.0566	100.3744	61.1884
Si4O8	557118	1.8221	106.5286	65.6414
Si4O8	557134	1.9597	100.426	61.2029
Si4O8	557837	1.6333	100.4018	61.7687
Si4O8	557873	2.325	87.8675	53.3257
Si36O72	558025	3.6165	72.6983	43.9237
Al10P10O40	558056	3.5487	67.9138	38.3985

Ca2H12C4O14	559469	2.0695	79.636	47.292
Ca4Zr2Si8O24	560339	1.9797	112.7073	71.7862
Si4O8	562490	2.5528	74.2904	44.9477
Mg4Al4P4O20	6596	1.9176	113.4807	64.7882
C60	667273	2.8287	144.9412	133.1389
Al18P18O72	667310	4.9286	62.4525	35.1768
Si4O8	7029	1.8398	97.3968	59.8437
Be8P4H20O28	705909	1.7619	82.193	46.4051
Zr2P4O20	1178611	1.8578	68.3414	32.7592
P8Pb4O36	1179996	1.6398	70.8373	30.7388
Ca6S6O27	1198084	1.8048	79.3272	38.4177
Si96H96O144	1199781	2.1169	64.1149	33.7842
P4O10	2452	1.6503	89.8442	48.331
Al10F30	555026	1.9424	105.9813	48.1805
Na8H16S12O48	707470	1.5608	69.0749	33.1916
Al4Si4H8O18	720262	1.8093	69.5427	33.8302
Rb4H12S8O32	733612	1.5193	61.1723	27.4645
Na4H32O20	733637	1.5737	70.5402	40.4931
Na8Zr4Si12O40	1180329	1.5477	78.5299	49.0968
Na18Al4H54O42	1199831	1.7069	70.1846	41.8299
Si96O192	1200292	4.1843	68.6359	41.3496
Zr4P8O32	1201586	1.9063	83.8293	41.642
Si16O32	17279	4.2803	64.8728	38.946
P8S2O14	29081	1.7305	57.1549	29.6228
H16S4O20	29095	1.6178	73.7802	35.4133
Mg8Si4O16	5392	1.55	130.4516	75.3447
Na4Ca2P8O24	541522	1.7631	94.113	57.7265
Si48O96	554682	4.2746	68.3281	41.1516
Si4O8	555235	2.1389	91.1974	55.831
Si32O64	560336	4.8797	65.444	39.3162
Si28O56	560708	4.0859	72.8026	44.0004
Si16O32	600037	4.4937	64.853	38.921
Si28O56	662706	4.1109	72.9716	44.1096
Al24P24O96	667363	4.7642	59.8566	33.6944
Si56O112	667371	4.0912	75.6863	45.8262
Si56O112	667373	4.0839	72.96	44.094
Si56O112	667376	4.1044	74.3374	44.9659
Si56O112	667377	3.8372	74.3766	44.9807
Si28O56	667383	4.0606	71.3102	43.0569
Si48O96	667448	3.7861	69.0673	40.6434
Be4P4H12O20	697592	1.7896	92.4624	50.375
Ca2P8H4O24	697657	1.741	88.6793	43.6954
Si24O48	733790	2.0841	95.7164	58.7558
Al8Si12H12O48	734142	2.0034	84.2014	42.9641
Al4Si4O14	1103424	2.0609	90.3291	52.654
Mg6Si4O18	1192605	2.1303	93.6423	48.7295
Si12O24	17909	4.3282	56.2724	33.5417

Si12O24	18280	4.1782	66.4054	39.9439
Be6P4H8O20	24674	1.5621	115.6308	65.7492
Na8Si7O18	29176	1.7522	98.3379	70.582
Si32O64	553945	4.4505	69.1844	41.6923
Si16O32	556262	4.5261	66.0848	39.7119
Al24P24O96	557362	4.7373	60.0216	33.8036
Si48O96	558947	4.2392	66.5552	40.0328
Si24O48	559360	4.126	67.2656	40.4841
C4	568286	1.6184	173.5041	165.4812
C4	568363	1.6883	169.7957	161.7241
C8	579909	2.0841	148.9001	130.8585
Mg4Al8Si10O36	6174	2.7259	111.6488	67.6571
Si22O44	680204	3.7582	83.1786	50.6129
Zr4P8H8O32	707540	1.8893	91.6507	44.3192
Na12H4S8O32	707941	1.8566	64.6555	34.1636
P4H8Pb2O16	733929	1.7673	81.3785	35.1462
NaPF6	10474	1.7259	56.2686	23.0333
Na16Mg16P48O144	1196753	1.5663	90.0397	52.3433
Mg8P8O32	1197696	2.2336	92.355	46.6681
Mg4H24C4O24	1199041	1.6125	86.9471	49.4795
Ca6Si6O19	1199535	1.9427	90.9066	58.467
Ca12Al8O48	1200701	1.579	60.1543	27.8301
H8S4O16	24172	1.6112	68.319	31.0928
Mg5H2O6	30241	1.5091	125.7158	64.5018
Mg6H4O8	30243	1.5248	117.9681	60.2901
Mg2H2O3	30244	1.5531	108.4294	56.3284
Na4P8H20O32	505701	1.9481	73.4658	39.4129
Si24O48	542814	2.1192	90.6664	55.4816
Al24P24O96	5440	2.8261	74.2328	42.0867
Si32O64	554755	4.0908	55.6292	33.1174
Al6P6O24	557915	2.5799	78.0987	44.3748
C120	568028	2.3966	188.8559	173.2436
Al12P12O48	5859	4.8274	58.5013	32.9172
C120F36	647169	2.4496	118.795	84.712
Si8O16	669426	1.755	99.1466	60.9631
C60	680372	2.6864	174.1492	160.9379
Zr2P4H12O20	697914	2.0054	82.959	41.4814
Si16O36	1179275	2.0328	79.3598	45.3948
Zr2P4C2O12	1189787	2.056	94.0972	49.2578
Mg12S8O48	1199206	1.687	73.5892	30.5669
Be12Si4O24	1202186	1.6892	127.638	83.1121
Al8P4O32	1204519	1.7229	78.7648	38.6918
Mg48Si34H62O147	1205288	1.7761	109.1497	58.4155
C4O8	556034	1.525	74.2676	46.01
Si18O36	556591	3.7051	65.039	39.0497
Be4P8O24	560521	1.5833	101.8549	58.6297
Al32P32O128	683883	3.6433	61.7129	34.7616

Sn4H4C8O12	697873	1.5035	92.764	44.645
Na8Si12H8O32	698025	1.6327	80.4305	49.0236
Al4Si4H8O18	698132	1.9629	118.8126	64.537
Al6P4H28O30F6	698160	1.7871	103.437	52.7323
Mg3P2H44O30	698183	1.9447	83.1685	45.9662
Na4H40C2O26	699482	1.7975	70.3432	42.2047
Ca16Si12H8O44	1197100	1.5127	101.8775	60.3208
MgAl2S4	16755	1.8563	55.6577	29.6067
Mg6Si4O18	1193906	2.1077	89.2206	46.0819
Na8Zr2C16O38	1197594	1.8003	71.0942	47.8926
Na3H5C4O8	555083	1.7748	64.0714	41.6487
P6H18Pb6C6O18	559039	3.0298	58.8439	25.1866
H16Pb4C8O24	600174	1.6622	85.6421	40.4266
Na12Al4H40C24O68	698350	1.8244	73.9305	46.1767
Na8P4H60O44	708993	1.6465	68.7855	40.507
Na6H18C12O24	738668	1.767	60.2524	38.7133
AlF3	8039	1.9419	109.219	49.7358
C4O8	1182086	1.8581	56.8452	35.1048
Bi4C12O36	1182384	2.0581	69.0986	34.8879
Ca12Si12O38	1196268	1.9175	91.9472	59.1952
Si72O116	1199711	4.5746	65.8493	38.0151
Ca6Si6O19	1202163	1.8826	92.2667	59.4104
Mg16Si24O72	1203861	2.0171	103.5733	57.6906
Si72O144	1205213	4.657	73.1211	43.8993
O8	12957	1.6294	50.4455	20.6416
Na4Bi4P16O48	23621	1.8488	101.1797	48.9744
H4C8O8	23680	1.8491	98.2463	59.7655
H12C6O6	23683	1.5541	82.9273	52.0266
Ca8H16C16O32	23685	1.5139	78.014	48.1767
Rb8H24C16O32	23688	1.6744	52.9199	31.0421
Be2Al2H10	23719	1.7737	54.8695	33.8081
Mg4H24C8O24	542785	1.7746	83.2282	48.4544
Na24Si32O76	554033	1.5847	83.0497	58.1772
Ca8P8H32O44	554174	1.7677	81.2221	43.443
Si16O32	554267	2.8616	78.8663	47.8328
H32C12O8	555128	1.5294	73.9132	48.8052
H40C20O20	555711	1.7321	85.5603	53.5956
H32C12S8	555848	1.606	51.4619	33.337
Si2Sn12O16	556100	2.1077	77.2068	29.0394
CaZr4P6O24	556440	1.6269	108.6425	58.6921
Al2P6H12O24	556596	1.6887	91.5241	46.1564
Al8P12H36C12O36	556858	3.699	56.2619	31.9549
Ca8H16C16O40	557769	1.5012	91.8469	56.0569
Si6Bi4O18	558672	1.8884	113.933	53.0166
C6F12	559432	1.607	66.9602	31.3129
Si32O64	560064	3.9762	57.1899	34.1082
Si18O36	560155	3.0027	69.0774	41.5823

Ca4H8C8O16	560210	1.5364	76.0158	46.867
Si24O48	561351	3.6915	74.4388	45.0306
Sn4H32C16O16	561405	1.6821	72.0054	41.5471
Si64O128	600054	4.0032	57.0896	34.0308
Na4H8C4S4O16F12	601202	1.7769	59.9323	27.2561
Na4H20C4O16	708984	1.8188	61.1702	38.7911
Na4H12C16S16O48F48	709432	1.9024	53.7147	22.2093
Ca6Si6H2O19	1183664	1.887	95.8426	58.4163
H24C142F8	1196283	2.7331	117.8458	92.838
C156F84	1196461	2.7331	115.0585	72.3696
Mg17Si20O60	1196569	2.0151	123.6737	72.3527
Mg20Si24O72	1198184	1.9951	121.8324	70.6998
Ca2Al4Si8O33	1200127	1.7782	83.9877	42.7742
Ca2Al4Si6H12O26	1203998	2.1177	89.8242	49.2712
Na4Ca4Al12Si18H32O76	1204100	2.0428	88.1849	49.6882
Si8Sn8H164C64O12	1205138	2.3596	53.0736	36.6062
Ca2Mg5Si8O24	1237404	1.8757	95.7754	56.0811
Rb8Be4H16S8O40	542884	1.5965	61.7316	29.9224
Si48O96	554946	3.9684	69.2075	41.707
Mg4Al4P4O20	555548	1.6695	117.2609	66.9827
C74F42	555948	2.6818	110.45	68.8002
C16O20F24	556192	1.6972	75.8289	38.1517
Si16H96C32	569770	1.934	53.3054	38.8439
Al6H18	570130	1.578	51.8863	29.9848
Mg8Si8O24	642210	1.5929	104.646	61.1382
C136F40	644802	2.7502	133.8555	96.4167
Al16P16O64	1019510	4.2019	65.8795	37.2124
Zr6F24	1194272	1.5447	131.3584	53.7555
Si20H114C38	1196075	2.6778	50.6492	36.8825
Sn2C48F40	1199905	1.9216	79.8241	43.764
C164F56	1200162	2.7854	127.9657	89.537
Si20H114C38	1202794	2.7255	50.078	36.5266
Al4H12O12	555462	1.5143	120.8609	67.0135
Si32O64	555521	4.6053	68.6782	41.3783
C8S4O16F16	555888	1.6864	59.6791	28.5057
P8H72C24	762352	1.7308	57.1334	40.6213
Si8H48C16	978595	1.895	63.9875	46.0899
Mg3Si4O13	1189710	1.514	107.1622	58.6943
Mg8P8C16O48	1197911	1.6698	92.0219	52.8727
Rb4H4C16O16	1200051	1.5551	66.3548	43.9942
Si16O32	1200685	3.0313	68.6737	40.9685
Si16H144C52	1202323	2.7476	55.5621	41.6297
Sn4C16O16	1202685	1.999	54.464	29.1383
Na8Mg8P8C16O48	1202863	1.593	86.5763	56.8524
Mg45Si32H58O138	1204533	1.8906	109.1474	58.4187
Zr4H32Pb4C24O64	1204872	1.7	98.9174	51.6809
Si4O8	545719	1.7406	96.1534	58.0222

Si16O32	553993	3.6449	56.9395	33.8606
Si16O32	554175	3.7407	63.6485	37.836
Si16O32	554258	3.1421	72.1387	43.5823
Si8O16	554397	1.646	102.1338	58.0058
Si12O24	554498	4.446	77.3247	42.6283
Si8O16	554543	3.1227	73.2524	44.27
Si8O16	554665	2.4119	91.0484	54.4795
Si16O32	555165	1.642	111.095	68.559
Si12O24	555355	3.7189	89.7161	50.0363
Si8O16	555411	2.2354	102.2829	58.8856
Si24O48	555497	2.1244	127.7356	77.1448
Si8O16	555544	2.5176	85.2423	50.975
Si16O32	555556	2.7124	81.5895	49.6145
Si8O16	555676	2.1072	98.5383	58.8285
Si16O32	555700	2.7608	74.9721	45.3885
Si12O24	555823	3.6221	83.6989	46.6948
Si12O24	556044	3.7994	55.4269	28.3895
Si48O96	556257	2.9729	69.6813	40.3183
Si12O24	556464	2.1899	125.037	75.5402
Si12O24	556537	2.3236	99.6642	59.5486
Si16O32	556564	1.8584	101.6377	54.7796
Si6O12	556812	1.7706	137.8623	83.0817
Si12O24	556963	3.712	100.3675	60.736
Si12O24	557004	2.9192	72.0428	43.497
Si16O32	557017	3.0122	104.6871	60.5401
Si48O96	557024	3.9282	62.2444	37.121
Si6O12	557194	2.2164	73.7624	38.8419
Si24O48	557211	4.7606	63.7689	38.2641
Si8O16	557264	2.4095	87.5236	53.4489
Si8O16	557465	1.7065	111.1465	68.6533
Si24O48	557591	3.6305	79.9773	48.2718
Si12O24	557723	2.0727	92.3906	54.8343
Si12O24	557771	4.027	52.9995	29.9263
Si48O96	557814	3.9478	81.677	45.9756
Si12O24	557881	3.0315	70.05	42.2343
Si8O16	557930	1.9581	101.9519	62.5835
Si40O80	558115	4.314	68.768	41.4412
Si8O16	558200	2.3446	106.4647	65.1778
Si16O32	558301	3.4568	62.5404	36.7511
Si12O24	558351	3.0565	69.7836	42.067
Si8O16	558366	2.0612	97.5977	54.513
Si24O48	558374	2.2576	89.137	54.3456
Si12O24	558564	4.4542	67.9819	40.6958
Si24O48	558569	2.7026	97.9243	59.6864
Si12O24	558598	3.9155	58.0115	28.1262
Si12O24	558891	1.7474	101.4717	62.4222
Si48O96	558931	4.0778	76.6111	45.1693

Si8O16	559313	2.3682	78.8217	47.8432
Si16O32	559347	1.7715	110.6548	68.3043
Si6O12	559605	2.7441	80.58	47.7645
Si8O16	559860	1.7311	92.2695	55.4952
Si6O12	559928	3.3484	60.2038	35.957
Si16O32	560152	1.7446	103.1155	62.8328
Si8O16	560527	2.8244	71.7086	43.3021
Si16O32	560754	2.4632	101.7355	58.7312
Si12O24	560836	3.8505	69.9295	41.2354
Na6Si6Sn2O18F2	560937	1.8076	86.7564	53.4494
Si8O16	560954	1.738	89.2525	53.5391
Si28O56	561181	4.5536	58.0316	34.6329
Si8O16	561291	2.3958	100.0576	60.5234
Si48O96	561301	3.309	101.4199	58.8098
Si12O24	561488	4.5674	63.4667	36.3746
Be6F12	561543	3.7303	53.6174	28.4626
Si16O32	572283	2.3322	124.4153	75.9572
Si8O16	638033	1.575	94.2019	53.1556
Si8O16	638038	2.7808	69.4367	36.6004
Si12O24	638049	2.08	117.0672	57.1157
C32S8O16	638445	1.8264	82.2686	55.5029
Rb8C8S8O24F24	672209	1.7476	51.0969	22.1948
Si8O16	683947	2.789	94.0662	56.7874
Si48O96	683952	3.9333	84.2718	47.3433
Si48O96	683953	2.8074	78.6804	43.4237
C72F36	683965	2.7183	123.572	79.5423
Rb2C2S2O6F6	6858	1.5656	50.8468	22.1066
Mg6O6	1101930	1.7432	75.5493	43.038
Al6H18	1183246	2.2975	51.8064	29.9777
Si6O12	1188220	3.8176	65.7437	39.5282
C20	1188817	1.6114	268.0286	242.6094
Mg12O12	1190533	2.6607	81.945	46.9768
Mg12O12	1190649	1.9576	82.7006	47.0174
Mg12O12	1191789	2.211	70.8267	40.048
H16S8O40	1197484	1.553	65.6886	28.4141
Al4H84C12S12O60	1201938	1.7101	71.8518	39.1655
Si46	971662	2.1427	56.1771	37.5016
C12	1095534	1.977	142.7094	123.2172
C12	1095633	2.1459	115.9631	101.0928
Bi4S6O24	1195213	2.1159	78.5316	33.3538
Si40	1196961	2.6303	55.2177	36.8749
Na12Mg4P12H96O88	1197608	1.7475	71.8664	41.4056
Na2MgH32S2O24	1199634	1.6786	67.7501	37.7109
Si82	1199894	2.25	55.3024	36.7304
CaSnP6H12O12	1200443	1.8231	60.0875	27.6085
Si41	1200830	2.4278	54.1159	36.1115
Si232	1201492	2.6591	54.3042	36.1811

Si58	1202745	2.6097	52.6458	35.1163
Si68	1203790	2.8304	55.2518	36.9803
Si106	1204046	2.3547	53.8921	35.6927
Si46	1204627	2.5549	54.69	36.5024
Na8Al8Si16O48	1020661	2.0683	74.3757	49.6346
Sn12O16	1179450	1.8405	64.7998	22.3677
Na8H72S4O36	1180434	1.8097	61.2492	35.5305
Ca8P8H8O32	1194747	1.564	93.4393	49.7726
Si34O74	1194828	3.9397	68.2276	39.6576
Zr4S4O24	1197006	1.8857	101.4354	46.8729
ZrP2H2O6	24030	1.5304	91.0867	42.5942
Mg3P2H16O16	24638	1.5291	101.475	54.8747
Na6Mg4P10O32	555005	1.6265	86.7643	53.1396
Zr8P32O96	557909	1.8126	113.6724	60.369
Al6F18	559871	2.5557	99.9044	45.3501
Ca4Al8Si8O32	758658	1.6294	115.4419	74.2171
Mg6P4H32O32	766572	1.9704	96.5545	52.2304
Al8O24	1182911	1.6522	78.4499	35.8241
Al8Si8Pb4O32	1194510	1.672	120.1838	62.875
Mg12H48C48O48	1194668	2.027	79.7864	51.0625
Na8Zr4Si24H24O72	1194692	1.897	95.7399	54.0935
Al16Si16Pb8O64	1195073	1.6665	119.1199	62.2967
C24F40	1195459	1.664	82.0156	39.7602
C24F40	1195560	1.6141	84.0752	40.7526
Mg4H48C12S4O28	1204393	1.948	74.1329	42.9073
Mg12H24C24O48	555000	3.3347	54.242	31.9173
Ca4C8O16	558543	2.3594	74.6185	51.0084
Sn4S4O16	645709	1.5886	76.5123	32.813
Sn4S4O16	645740	1.7122	66.054	28.3141
Sn4S4O16	645774	1.5396	76.9148	32.9921
Pb2C8O8	1179835	1.6972	85.5404	48.2138
Mg2C8O12	1191802	1.8172	70.5981	40.5943
Ca4Al8Si12O42	1194616	2.1574	92.284	55.309
Ca2Al4Si6H8O24	1194858	2.0175	90.4905	49.555
Ca2Al4Si6O20	1195158	2.1089	98.148	60.7146
H24Pb4C8S8O28	1195902	1.7448	65.403	28.4646
Na6Mg6Al6H108C36O126	1198421	1.8843	73.6005	43.4498
Al4H12C15O31	1199608	2.0096	82.6151	47.8289
Bi2H50C16S12O24	1200655	1.9704	60.233	32.1235
P8O12F16	1200699	1.7623	58.3964	26.4654
Si36O72	558326	3.7407	71.6051	43.2317
Si18O36	560998	3.7538	70.221	42.3528
C136O2F40	645279	2.7453	131.2274	93.9592
C140F60	645316	2.73	122.2509	81.4518
Mg2P4H32O20	758660	2.2417	69.9031	37.7634
Mg2Si2H24O12F12	759312	1.8473	72.0413	35.2431
Si8H96C32	978526	1.9781	75.4785	55.3466

Al4P12O36	1019377	2.3736	90.2729	50.3639
H24C8S8O24	1199075	1.8095	51.7252	25.9895
C216F120	1199402	3.0869	109.5343	68.694
Ca2P4H16C4O12	1203091	1.744	75.8204	44.2238
Al2P2O8	545974	2.3489	72.4042	41.0225
Si16O32	556454	3.8932	73.3932	44.3837
Zr2P4O14	557754	1.9162	96.1312	50.5348
Si12	1095269	2.0518	60.539	39.906
Ca4Al8Si8H20O42	1197037	1.911	100.6508	55.7211
Bi2H36C6S6O36F18	1199850	2.263	62.4037	27.6474
Mg4P16O44	15437	1.9283	95.3109	50.6188
Na8Ca2P12O36	542280	1.6264	83.3161	51.6526
Al2P2O8	5724	2.029	83.4797	47.5597
Na8Mg8P24H64O80	849748	1.8034	73.1945	38.7882
Al4S8O48	1204923	2.042	56.5524	25.3535
C4O6F12	29479	1.5421	60.7377	26.7147
Sn4O2F10	29590	1.5373	76.0773	24.99
Al8Bi4S16	557737	2.2441	50.8339	23.7411
Al6P18O54	559996	1.6959	107.581	59.9582
Si32O64	560941	4.7633	60.7906	36.3847
Na2Sn8P6O24	6226	1.7373	80.6894	33.9893
Al8H40S12O68	757796	1.8847	92.777	45.1165
Al8H64S12O80	850293	1.8153	86.3215	43.5419
Na4Mg2P8H24O36	1197995	1.8631	76.5852	41.2251
C22F14	1588	2.1425	83.0876	49.3892
Mg2Al2H8O4F10	24142	1.7688	95.2621	42.6929
P16O72	31272	1.7227	65.0098	30.559
Si32O64	557894	4.6144	65.558	39.3749
Sn10P4O20	560715	1.8892	75.4696	31.0086
C24O16	561848	1.583	66.2307	46.0305
Si64O128	600080	4.1295	55.2613	32.8703
C140	683919	2.7246	149.1512	137.5675
Mg6Si4O18	1194460	2.112	89.4631	45.8429
C16O24F16	31306	1.7912	73.7404	38.9818
Si24O48	556654	2.5294	84.6157	51.5658
Ca8P12O38	559537	1.5738	100.5664	58.6645
Zr4P4O18	560358	1.7954	131.6344	70.7935
Al4P6H22O26	605108	2.1851	86.5574	45.1956
Na8Si4S24O84	1020159	2.1066	65.5486	30.9361
H16S4O20	1181245	1.5205	82.0565	39.0732
Na4Ca4P4H72S4O48	1202262	1.9192	71.625	40.6797
Si6O12	559550	3.3465	75.9234	45.9979
Rb2Mg2P2H24O20	604725	1.6225	72.6538	41.2384
PHPb2F10	865973	1.5016	75.6155	23.5974
Ca4S12O40	1019581	1.7084	78.1489	35.224
Mg2S4O14	1020122	1.9335	80.3192	37.4076
Rb4Si2S12O42	1020711	1.5251	64.6465	30.0015

Bi4S12O42	1200203	1.6529	75.3262	33.7047
Na12Sn6S36O126	1202012	2.0467	63.9275	28.1517
Si48O96	1203655	4.2907	66.4879	39.9223
Rb4H4S8O28	1195562	1.6974	57.0615	24.8841
Al4Pb2S8	1103722	1.6108	56.6345	26.1656
Mg2P2S6	675651	1.6738	50.2195	22.7025
